# Pediatric multicellular tumor spheroid models illustrate a therapeutic potential by combining BH3 mimetics with Natural Killer (NK) cell-based immunotherapy

**DOI:** 10.1038/s41420-021-00812-6

**Published:** 2022-01-10

**Authors:** Vinzenz Särchen, Senthan Shanmugalingam, Sarah Kehr, Lisa Marie Reindl, Victoria Greze, Sara Wiedemann, Cathinka Boedicker, Maureen Jacob, Katrin Bankov, Nina Becker, Sibylle Wehner, Till M. Theilen, Steffen Gretser, Elise Gradhand, Carsten Kummerow, Evelyn Ullrich, Meike Vogler

**Affiliations:** 1 Institute for Experimental Cancer Research in Pediatrics, Goethe-University Frankfurt, Frankfurt am Main, Germany; 2 Children’s Hospital, Goethe-University Frankfurt, Frankfurt am Main, Germany; 3 Experimental Immunology, Goethe-University Frankfurt, Frankfurt am Main, Germany; 4 Dr. Senckenberg Institute of Pathology, Goethe-University Frankfurt, Frankfurt am Main, Germany; 5 University Cancer Center Frankfurt (UCT), University Hospital Frankfurt, Goethe-University Frankfurt, Frankfurt am Main, Germany; 6 Department of Pediatric Surgery and Pediatric Urology, University Hospital Frankfurt, Goethe-University Frankfurt, Frankfurt am Main, Germany; 7 Department of Pediatric and Perinatal Pathology, Dr. Senckenberg Institute of Pathology, Goethe-University Frankfurt, Frankfurt am Main, Germany; 8 Department of Biophysics, Center for Integrative Physiology and Molecular Medicine, School of Medicine, Saarland University, Homburg, Saarland Germany; 9 Frankfurt Cancer Institute, Goethe-University Frankfurt, Frankfurt am Main, Germany; 10 German Cancer Consortium (DKTK) partner site Frankfurt/Mainz, Frankfurt am Main, Germany

**Keywords:** Paediatric cancer, Tumour immunology

## Abstract

The induction of apoptosis is a direct way to eliminate tumor cells and improve cancer therapy. Apoptosis is tightly controlled by the balance of pro- and antiapoptotic Bcl-2 proteins. BH3 mimetics neutralize the antiapoptotic function of Bcl-2 proteins and are highly promising compounds inducing apoptosis in several cancer entities including pediatric malignancies. However, the clinical application of BH3 mimetics in solid tumors is impeded by the frequent resistance to single BH3 mimetics and the anticipated toxicity of high concentrations or combination treatments. One potential avenue to increase the potency of BH3 mimetics is the development of immune cell-based therapies to counteract the intrinsic apoptosis resistance of tumor cells and sensitize them to immune attack. Here, we describe spheroid cultures of pediatric cancer cells that can serve as models for drug testing. In these 3D models, we were able to demonstrate that activated allogeneic Natural Killer (NK) cells migrated into tumor spheroids and displayed cytotoxicity against a wide range of pediatric cancer spheroids, highlighting their potential as anti-tumor effector cells. Next, we investigated whether treatment of tumor spheroids with subtoxic concentrations of BH3 mimetics can increase the cytotoxicity of NK cells. Notably, the cytotoxic effects of NK cells were enhanced by the addition of BH3 mimetics. Treatment with either the Bcl-X_L_ inhibitor A1331852 or the Mcl-1 inhibitor S63845 increased the cytotoxicity of NK cells and reduced spheroid size, while the Bcl-2 inhibitor ABT-199 had no effect on NK cell-mediated killing. Taken together, this is the first study to describe the combination of BH3 mimetics targeting Bcl-X_L_ or Mcl-1 with NK cell-based immunotherapy, highlighting the potential of BH3 mimetics in immunotherapy.

## Introduction

Although considerable progress has been made in the treatment of childhood cancer, refractory disease is still associated with a poor prognosis. Amongst the most common forms of extracranial solid tumors in children, neuroblastoma and rhabdomyosarcoma (RMS) display particularly poor outcomes in the high-risk patient groups, highlighting the need for improved treatment strategies [1, 2].

One approach in the treatment of cancer is the direct induction of apoptosis in tumor cells. Apoptosis can be initiated by the ligation of death receptors on the cell surface, or, alternatively, by the mitochondrial release of cytochrome c, which is controlled by the Bcl-2 proteins [3]. While Bcl-2 is mainly expressed in lymphoid malignancies, the related proteins Bcl-X_L_ and Mcl-1 are frequently amplified and overexpressed in solid tumors including pediatric cancers [4, 5]. Several compounds have been developed that bind and neutralize the antiapoptotic Bcl-2 proteins. Thereby, these inhibitors antagonize the antiapoptotic Bcl-2 proteins in a similar manner as BH3-only proteins, and are thus called BH3 mimetics. With ABT-199 (venetoclax), the first BH3 mimetic selectively inhibiting Bcl-2 has been approved and is transforming the treatment of lymphoid malignancies like chronic lymphocytic leukemia [6, 7]. Selective and potent inhibitors of Mcl-1 and Bcl-X_L_ have also been identified, some of which are currently being investigated in clinical trials [5, 8, 9]. The potential of BH3 mimetics targeting Bcl-X_L_ or Mcl-1 has been underscored by multiple studies showing their apoptosis-inducing capacities in many malignancies either alone or in combination with each other [10–15].

However, the anticipated toxicities of these compounds on nonmalignant cells raise questions about their safe use in humans. Bcl-X_L_ is an essential antiapoptotic protein in mature platelets, and hence inhibition of Bcl-X_L_ causes thrombocytopenia [16], which has been identified as dose-limiting toxicity in clinical trials with the dual Bcl-2/Bcl-X_L_ inhibitor ABT-263 (navitoclax) [17]. Deletion of Mcl-1 in mice is embryonically lethal and several lymphoid lineages depend on Mcl-1 for survival [18]. As Mcl-1 plays an essential role in cardiac muscle cells [19], a clinical trial currently ongoing for the selective Mcl-1 inhibitor AMG-376 (NCT03465540) has been placed on hold to evaluate cardiac toxicity. In light of these safety concerns, novel strategies are urgently required that allow safe usage of BH3 mimetics at lower non-toxic dosages.

To improve the translation of novel cancer treatment options, better in vitro models for cancer are required, reflecting more faithfully the characteristics and complexity of the disease (e.g., hypoxic microenvironment, drug penetrance). To this end, 3D spheroid or organoid cultures have been developed as useful tools for preclinical cancer research [20]. Growth in spheroids is achieved by the addition of extracellular matrix/scaffold components and/or by culture methods that remove the adhesive properties of the culture dish, thus forcing the cells to aggregate. Aggregation and growth in spheroids enable cell-cell and cell-matrix interactions and allow investigations with infiltrating immune cells [21, 22].

With the clinical use of engineered T cells, cell-based immunotherapies are emerging as powerful anti-cancer therapeutics. Besides cytotoxic T cells, also Natural Killer (NK) cells can be used to eliminate tumor cells [23, 24]. NK cells attack target cells either by perforin- and granzyme-containing secretory lysosomes, or by death ligands, i.e. Tumor necrosis factor (TNF)-related apoptosis-inducing ligand (TRAIL) or FasL, activating a variety of cell death pathways [25, 26]. Of note, granzyme B has been shown to engage the mitochondrial apoptotic pathway but also to directly induce caspase activation, highlighting the diversity of interactions between NK cells and the apoptotic machinery within target cells [27, 28]. The cytotoxic activity of NK cells is balanced by activating or inhibitory receptors on their surface. A major advantage of NK cell over T cell-based immunotherapy, is the MHC class I nonrestricted cytotoxicity, by which evasion strategies, i.e., downregulation of MHC class I molecules by malignant cells, can be circumvented. [29]. A promising strategy is the application of allogeneic NK cell preparations upon ex vivo stimulation with activating cytokines, such as interleukin (IL)−2, −15 or −21 [30–32].

In this study, we investigated whether BH3 mimetics could serve as sensitizers for NK cell-mediated attack of cancer cells. Thereby, we hypothesized that subtoxic concentrations of BH3 mimetics may be sufficient to prime cancer cells for NK cell-mediated cytotoxicity. To recapitulate the interaction of BH3 mimetics with NK cell-mediated killing we utilized novel spheroid co-culture models.

## Results

### Establishment of 3D spheroids for pediatric cancer

**Fig. 1:**
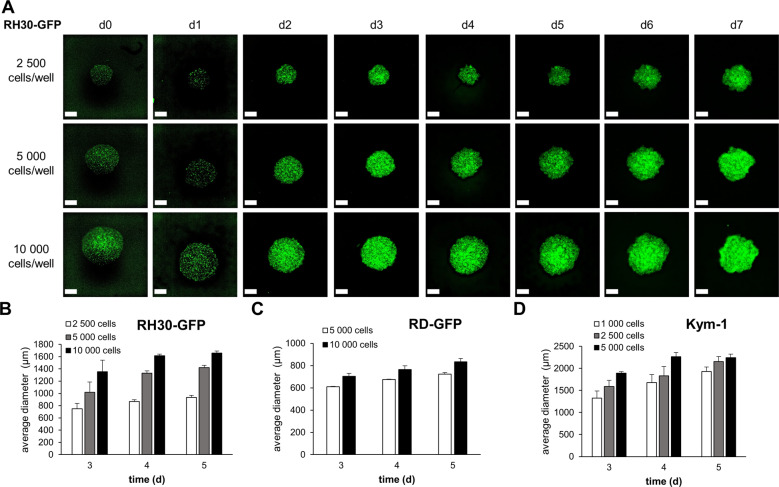
Spheroid formation in RMS cells. RH30-GFP cells were cultured in 96-well ultra-low attachment plates at different cell densities. To enable spheroid formation, plates were centrifuged at 1 000 g for 10 min directly after seeding. **A** GFP fluorescence signal of RH30-GFP spheroids was acquired for 7 days following spheroid formation. **B-D** Spheroid size was quantified at selected time points in RH30-GFP (**B**), RD-GFP (**C**), or Kym-1 (**D**) cells. Data shown are mean + S.D. (*n* = 2). Scale bar equals 500 μm.

To develop novel 3D models for pediatric cancer we adapted a protocol previously described [30, 33–35]. Aggregation of RMS spheroids formed rapidly after centrifugation and stayed stable for 2 weeks in culture (Fig. 1 and Supplementary Fig. 2). To investigate whether spheroid formation was a shared feature of pediatric cancer cells we extended our studies to osteosarcoma and neuroblastoma cells which all formed spheroids. However, we observed a mixed morphology of spheroids, with some cell lines like RD-GFP, U2OS, TE381.T, and UKF-NB3 forming round and compact spheroids, and others like RH30-GFP, MG63, and T174 forming loosely attached spheroids with diverse morphologies (Supplementary Fig. 3). The phenotype of the spheroids also influenced their size, as the round spheroids formed by RD-GFP cells were smaller and appeared to condense immediately after spheroid formation (Supplementary Figure 2 and 3, Video 1). To investigate whether spheroids can also be used as a culture system for primary patient-derived tumor cells we obtained neuroblastoma tissue and generated spheroids from the isolated cells. Although the morphology was diverse, these preliminary data indicate that also primary cells possess the ability to cluster and form scaffold-free spheroids (Supplementary Fig. 3).

### BH3 mimetics efficiently kill tumor spheroids

**Fig. 2:**
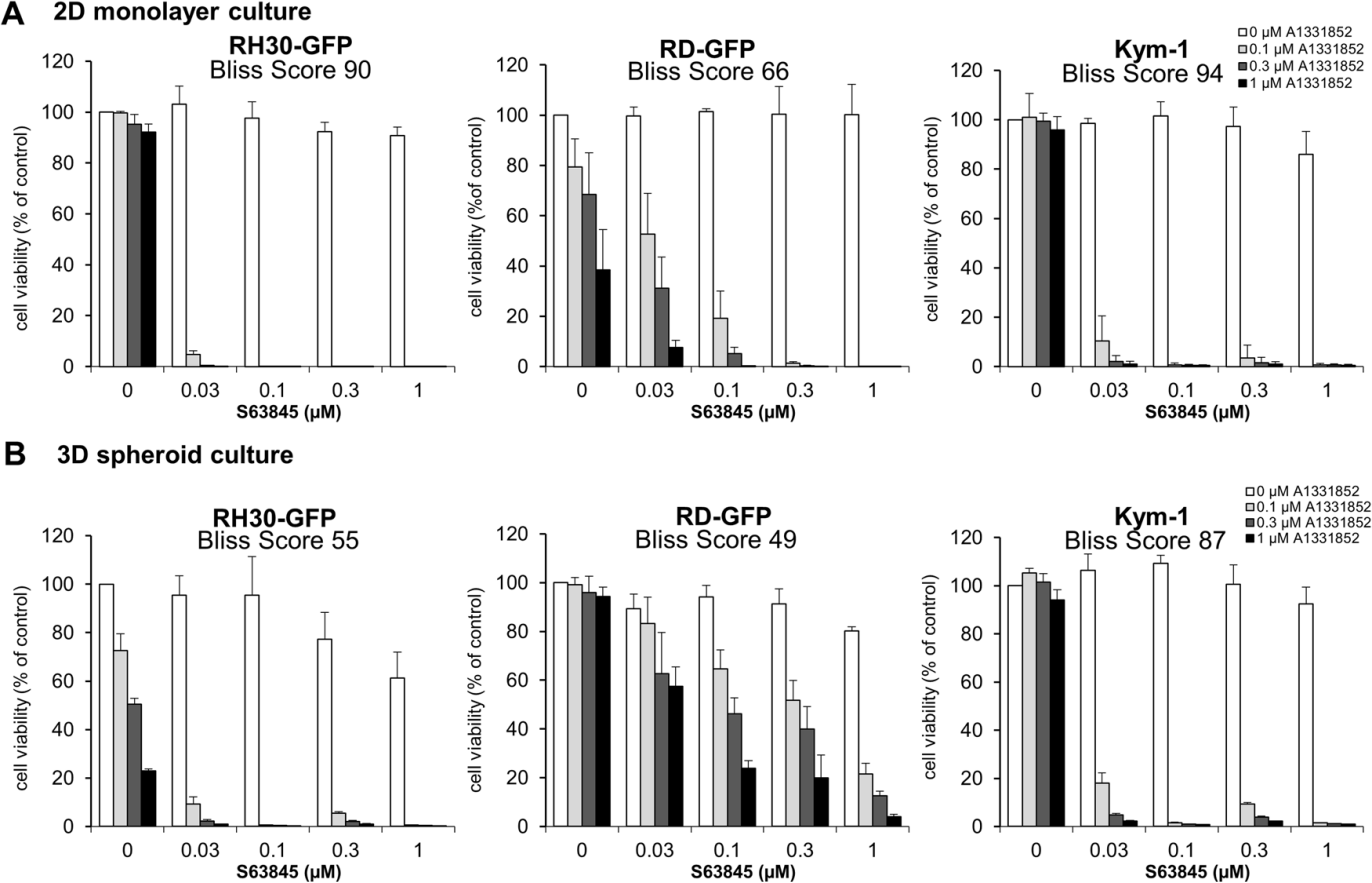
Cell death induced by BH3 mimetics in 2D and 3D cultures. **A** RH30-GFP, RD-GFP and Kym-1 cells were cultured as adherent monolayer cells (2D) before treatment with different concentrations of BH3 mimetics for 48 h. Viability was analyzed using Cell Titer Glo® assay. **B** RH30-GFP, RD-GFP and Kym-1 cells were cultured as spheroids (3D) for 3 days before treatment with different concentrations of BH3 mimetics for 48 h. Viability was analyzed using CellTiter-Glo® assay. Data shown are mean + S.D. (*n* = 3). Synergy was quantified using Synergyfinder and expressed as Bliss Score with a score >0 indicating synergy on a scale to 100.

**Fig. 3:**
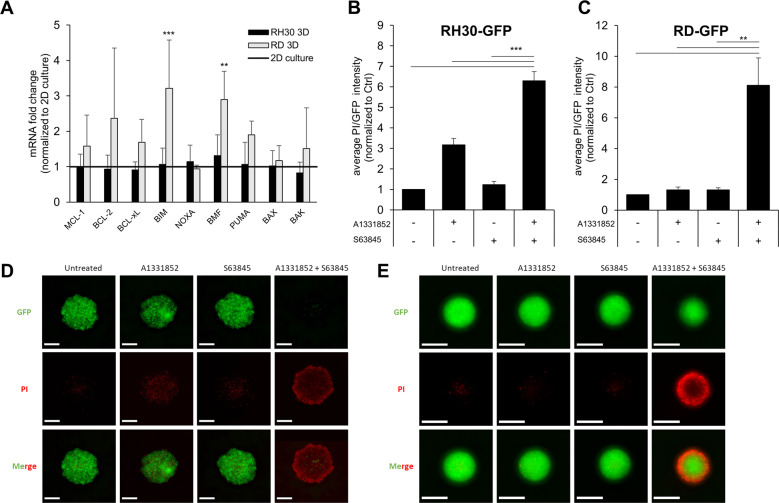
Microscopic analysis confirms cell death induced by BH3 mimetics. **A** mRNA expression levels of BCL2 family members were compared in 3D spheroids and 2D culture of RH30-GFP and RD-GFP cells by qRT-PCR. Data shown are mean + SEM (*n* = 4). **B**–**E** RH30-GFP or RD-GFP cells were cultured as 3D spheroids for 3 days before exposure to A1331852 (0.25 μM RD-GFP, 0.1 μM RH30-GFP) or S63845 (0.3 μM RD-GFP, 0.03 μM RH30-GFP) either alone or in combination for 48 h. Cell death was assessed by PI staining for 30 min before analysis. **B**, **C** PI and GFP fluorescence was quantified and expressed as the ratio of PI/GFP. Data shown are mean + S.D. (*n* = 3), **p < 0.01; ***p < 0.001. **D**–**E** Exemplary images are shown for PI and GFP fluorescence. Scale bar equals 500 μm.

To explore whether the cell line-based spheroids can serve as platforms for drug testing we investigated the response to apoptosis-inducing BH3 mimetics. We had previously shown that the Mcl-1 inhibitor S63845 and the Bcl-X_L_ inhibitor A1331852 synergized to induce apoptosis in RMS cells [11]. To compare the efficacy of these BH3 mimetics in standard 2D monolayer culture and 3D spheroid culture, we performed CellTiter-Glo® viability assay. While BH3 mimetics were able to reduce the viability of cells cultured in spheroids, the response to S63845 and A1331852 varied slightly between monolayer and spheroid culture (Fig. 2). In particular, the response to A1331852 as a single agent was increased in RH30-GFP spheroids but reduced in RD-GFP spheroids. Synergy, as assessed by calculation of the Bliss Score, remained robust in spheroids, with the round and compact RD-GFP spheroids being less susceptible to treatment with BH3 mimetics than loosely aggregated RH30-GFP or Kym-1 spheroids, indicating that the compact nature of the RD-GFP spheroids may be coinciding with increased apoptosis resistance. Comparison of BCL2 family gene expression in 3D spheroid culture with 2D monolayer culture indicated some alterations in the expression of BCL2 family genes in the RD-GFP spheroids, whereas the gene expression of the BCL2 family was not affected in the loosely aggregated RH30-GFP spheroids (Fig. 3A).

**Fig. 4:**
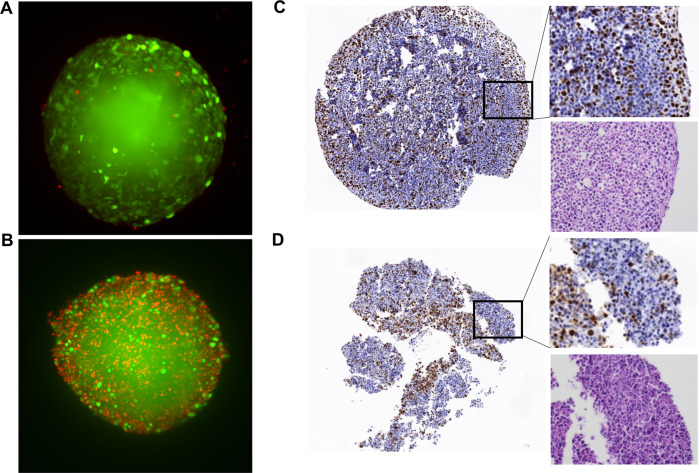
Morphological assessment of spheroids. **A**, **B** RD-GFP cells cultured as 3D spheroids were left untreated (**A**) or exposed to the combination of A1331852 (0.25 μM) and S63845 (0.3 μM) (**B**) for 48 h before PI staining and light-sheet microscopy. **C**, **D** RD-GFP cells were cultured as large spheroids containing 50,000 cells for 7 days and either left untreated (**C**) or treated with the combination of A1331852 (0.25 μM) and S63845 (0.3 μM) (**D**) for 6 h before formalin fixation and H/E staining (lower right side) or analysis of Ki-67 staining by immunohistochemistry (large image and upper right side).

Next, we investigated cell death induced by low concentrations of BH3 mimetics using PI uptake in the spheroids followed by live-cell imaging and plate-based microscopy. Throughout these experiments, we observed a prominent loss of GFP fluorescence upon induction of apoptosis. Therefore, cell death induced by BH3 mimetics in spheroids was quantified by the average PI versus GFP fluorescence intensity (for RD-GFP and RH30-GFP) or PI versus HOECHST intensity (for non-fluorescent Kym-1 cells) (Fig. 3B–E, Supplementary Fig. 4). All cell lines displayed increased PI uptake by combined treatment with A1331852 and S63845, confirming that simultaneous inhibition of Mcl-1 and Bcl-X_L_ induced cell death in tumor spheroids. However, while the RH30-GFP spheroids have completely lost their GFP fluorescence, the RD-GFP spheroids appeared to maintain a viable, apoptosis-resistant core. Light-sheet imaging confirmed the round and compact nature of RD-GFP spheroids. Treatment with BH3 mimetics increased the uptake of PI, with more PI-positive cells and loss of GFP on the outer ring (Fig. 4A, B). To investigate the role of proliferation in the spheroids we performed an immunohistochemical analysis of the RD-GFP spheroids. Staining of formalin-fixed spheroids with the proliferation marker Ki-67 demonstrated a high level of proliferating cells in the outer ring of RD-GFP spheroids. Combination treatment with A1331852 and S63845 induced cell death and reduced proliferation particularly in the outer ring, while proliferation appeared to be maintained within the core (Fig. 4C, D). Apoptosis induced in the outer ring was also clearly visible using H/E staining, which showed the occurrence of apoptotic bodies and loss of tissue cohesiveness upon treatment with BH3 mimetics. Taken together, these data suggest that the cells in the inner core of the compact RD-GFP tumor spheroids might be less susceptible to BH3 mimetic treatment, highlighting the need to identify treatment options capable of fully infiltrating complex tumor tissues.

### Tumor spheroids are attacked by NK cells

**Fig. 5:**
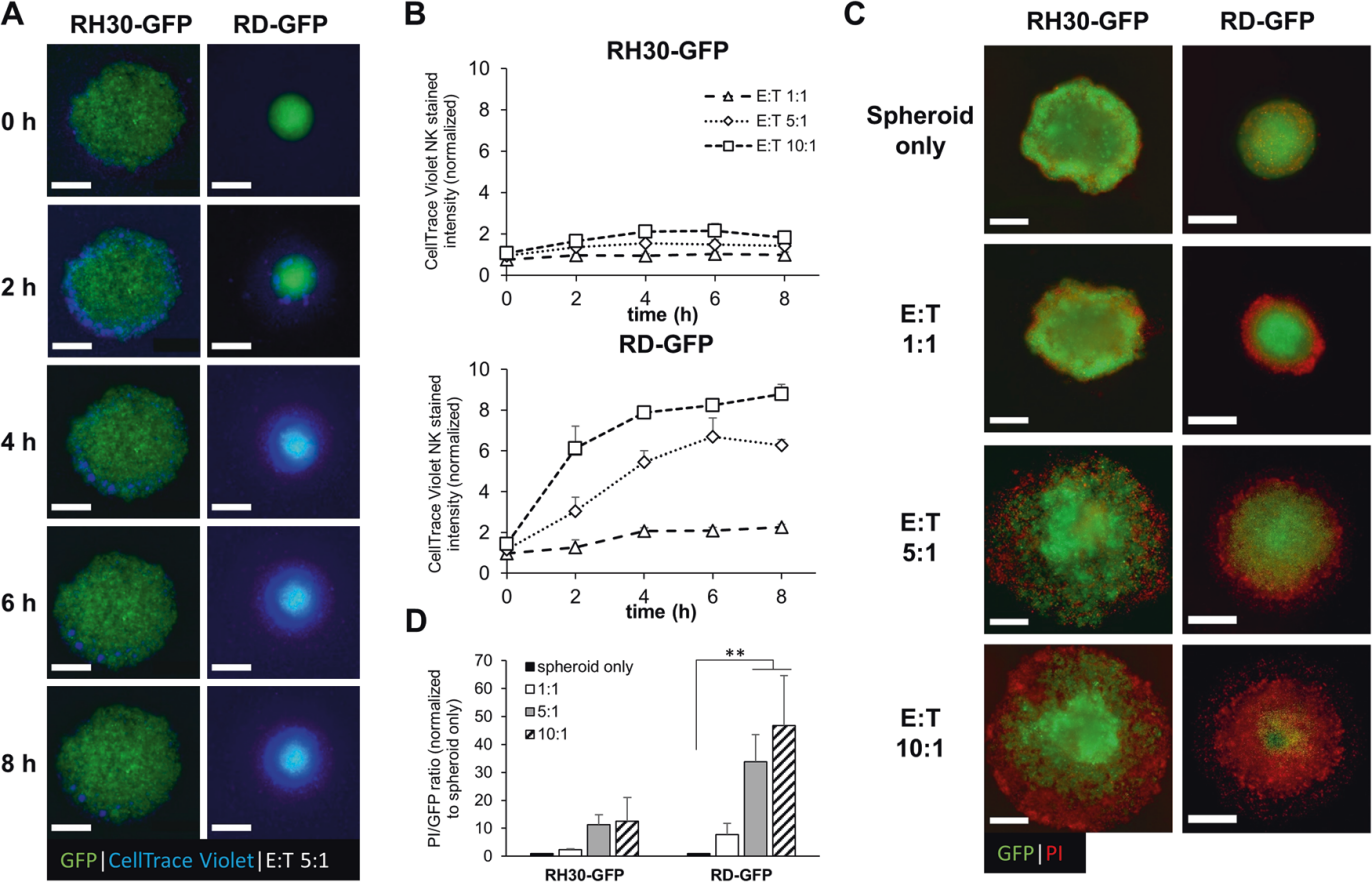
NK cells migrate into tumor spheroids to induce tumor cell killing. **A** RH30-GFP or RD-GFP cells were cultured as spheroids for 4 days. Activated NK cells were stained with CellTrace™ Violet and added to the spheroids at an effector to target (E:T) ratio of 5:1. Infiltration of NK cells into tumor spheroids was monitored by continuous live-cell microscopy for 8 h (see supplementary videos) and representative images for the indicated time points are displayed. **B** Modification of the E:T ratio influences the NK cell infiltration within the initial 8 h, measured by CellTrace™ Violet intensity within RH30-GFP or RD-GFP spheroids (mean + SEM, *n* = 3, normalized to only spheroids as background fluorescence). **C** NK cells are able to induce tumor cell killing in an E:T ratio-dependent manner. Selected images are displayed for the different E:T ratios after 5 days of NK cell addition. To visualize cell death spheroids were counterstained with PI. **D** Quantification of GFP and PI fluorescence intensity of RMS spheroids co-cultured with NK cells at different E:T ratios and calculation of the PI/GFP ratio as cell death indicator, exemplary images shown in (**C**). Data shown are mean + SEM (*n* = 5), **p < 0.01. Scale bar equals 500 μm.

An alternative approach to cancer therapy is the activation of immune cells to eliminate cancer cells. Therefore, we asked whether NK cells might be able to counteract apoptosis resistance observed within the spheroid core. Visualization of NK cells using CellTrace™ Violet, at different effector to target (E:T) ratios, showed that the NK cells rapidly attached to the tumor spheroids and formed clusters at the outer ring (Fig. 5A). In the loosely aggregated RH30-GFP spheroids, only few NK cells infiltrated the tumor spheroids, whereas the RD-GFP spheroids displayed high levels of infiltrating NK cells (Fig. 5A, B, Video 2, Video 3 and Supplementary Fig. 5A).

Next, we addressed whether activated NK cells may be able to attack the tumor spheroids. To this end, we added activated NK cells to the spheroids and analyzed PI uptake as a measure of cell death (Fig. 5C). Cell death induced by NK cells also coincides with the loss of GFP fluorescence (Supplementary Fig. 5B, C), and the ratio of PI versus GFP was used as a quantification of cell death. Activated NK cells were able to attack tumor spheroids as demonstrated by reduced GFP fluorescence and increased PI uptake (Fig. 5C, D). Thereby, not only the loosely aggregated spheroids formed by RH30-GFP cells, but particularly the compact tight spheroids formed by RD-GFP cells were efficiently killed by NK cells. Of note, the ability of NK cells to infiltrate and attack tumor spheroids was maintained and even increased in larger spheroids at a fixed E:T ratio, indicating that the surface area rather than the volume was the determining factor for NK cell attack (Supplementary Fig. 5D, E). Taken together, these data demonstrate the killing capacity of allogeneic NK cells and highlight the potential of NK cells for cancer therapy.

### The combination of BH3 mimetics and NK cells increases tumor cell killing

Based on these findings, we evaluated whether activated NK cells can be used in combination with BH3 mimetics to increase spheroid killing at subtoxic concentrations of BH3 mimetics. All tested BH3 mimetics A1331852, ABT-199 and S63845 did not induce cell death in activated NK cells at concentrations of < 1 μM (Supplementary Fig. 6A). Pretreatment of NK cells with BH3 mimetics did not alter their ability to attack RMS spheroids. Additionally, the migration of NK cells into the spheroids was not affected by pretreatment with BH3 mimetics, indicating that the functional activity of the activated NK cells was not affected by BH3 mimetics (Supplementary Fig. 6B, C).

**Fig. 6:**
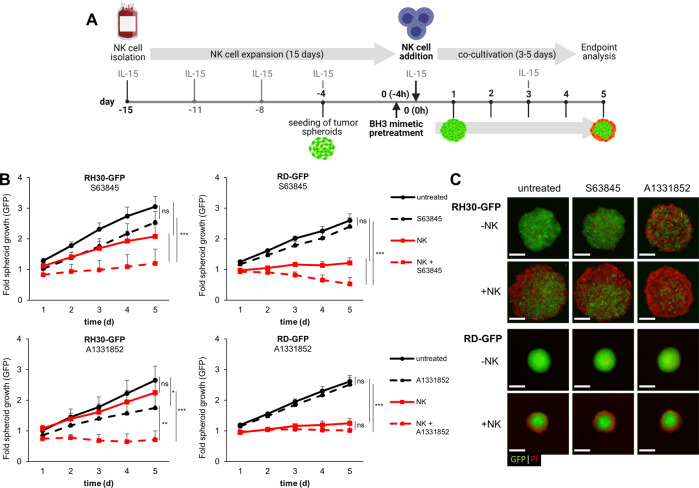
BH3 mimetics increase NK cell-mediated killing of tumor spheroids. **A** Experimental setup of combined BH3 mimetics and NK cell treatment. **B** Addition of the Mcl-1 inhibitor S63845 (0.3 μM) or the Bcl-X_L_ inhibitor A1331852 (0.3 μM) increases the NK cell-mediated effect (E:T ratio of 1:1) on spheroid growth inhibition. Data shown are mean + SEM (*n* = 5–7). ns: *p* > 0.05, *p < 0.05, **p < 0.01, ***p < 0.001. **C** Exemplary images of the combination of S63845 or A1331852 with NK cells showing increased cell death in tumor spheroids, indicated by PI counterstaining of GFP fluorescent RMS spheroids after 72 h of NK cell co-culture. Scale bar equals to 500 μm.

Next, we performed a combined treatment of tumor spheroids with BH3 mimetics and NK cells as outlined in Fig. 6A. Inhibition of spheroid growth by NK cells was further increased by the addition of either S63845 or A1331852 (Fig. 6B). In RH30-GFP spheroids both S63845 and A1331852 significantly increased the cytotoxicity of NK cells. In RD-GFP spheroids, the Mcl-1 inhibitor S63845 significantly enhanced growth control by NK cells, whereas the Bcl-X_L_ inhibitor A1331852 had no significant effect on NK cell cytotoxicity. To assess whether the combination of NK cells with BH3 mimetics also leads to more cell death, the spheroids were stained with PI. The images obtained indicated a more pronounced uptake of PI particularly in the RH30 cells (Fig. 6C). Of note, the Bcl-2 inhibitor, ABT-199, had no effect on NK cell-mediated killing of tumor spheroids in either of the cell lines tested (Supplementary Fig. 7). To investigate whether the sensitizing effect of BH3 mimetics is restricted to RMS spheroids or may also be applicable in other pediatric cancers, we used spheroids derived from neuroblastoma which were also efficiently killed by the combination of NK cells and S63845 (Supplementary Fig. 8). In contrast, spheroids derived from nonmalignant stromal cells did not display increased NK cell-mediated killing by the addition of BH3 mimetics, indicating some tumor selectivity (Supplementary Fig. 9)

**Fig. 7:**
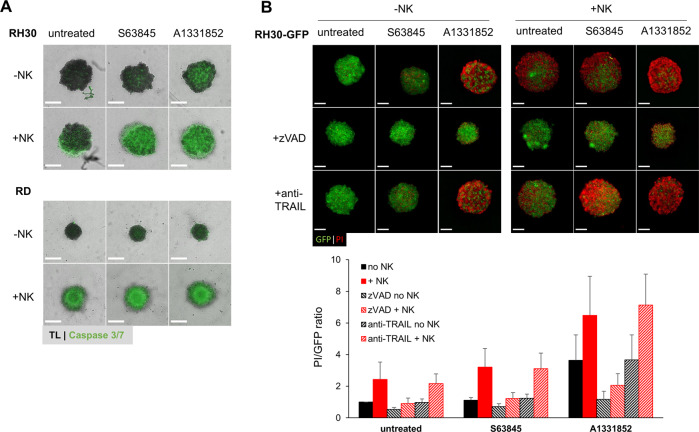
Caspase dependency of BH3 mimetic sensitization towards NK cell killing. **A** RH30 and RD wild-type spheroids were pretreated with either S63845 (0.3 μM) or A1331852 (0.3 μM) for 4 h, before addition of NK cells. After 24 h of NK cell co-cultivation, caspase activity was stained using a CellEvent™ Caspase 3/7 green detection substrate. TL: transmitted light channel. **B** To inhibit caspase activity or TRAIL, GFP expressing RMS spheroids were co-cultured with zVAD.fmk (50 μM) or anti-TRAIL blocking antibody (1 μg/ml) in combination with BH3 mimetics for 4 h before addition of NK cells (E:T ratio of 1:1). To visualize cell death, spheroids were counterstained with PI after 72 h of NK cell co-culture. Data shown are mean + S.D. (*n* = 3). Scale bar equals to 500 μm.

Finally, we aimed to unravel by what mechanism spheroids were killed by the combination of NK cells and BH3 mimetics. To investigate whether the combination of NK cells and BH3 mimetics induced cell death by apoptosis, we analyzed the activation of caspases using fluorescently labelled caspase substrate. The images obtained indeed indicated increased caspase activation by the combination of NK cells with BH3 mimetics (Fig. 7A). Next, we analyzed cell death by PI staining while simultaneously blocking caspases with the broad-range caspase inhibitor zVAD.fmk. In addition to, the inhibition of caspases we applied an anti-TRAIL antibody to block TRAIL signaling. The activity of this blocking antibody was confirmed in separate experiments using recombinant TRAIL as apoptosis-inducing agent (Supplementary Fig. 10). Interestingly, the addition of anti-TRAIL had no effect on tumor cell killing, suggesting that TRAIL on NK cells was not required for the killing of tumor spheroids (Fig. 7B). In contrast, addition of zVAD.fmk completely abrogated tumor cell killing by BH3 mimetics and NK cells, demonstrating that the NK cells cooperate with BH3 mimetics to induce caspase-dependent apoptosis in pediatric tumor spheroids.

## Discussion

By neutralizing the antiapoptotic function of Bcl-2 proteins, BH3 mimetics are powerful compounds to induce apoptosis in cancer cells. Therefore, they represent promising anti-cancer therapeutics, and their potential for the treatment of cancer has been highlighted by the clinical success of the selective Bcl-2 inhibitor ABT-199 in the treatment of leukemia. In contrast to hematological malignancies, BH3 mimetics have not yet achieved clinical benefit in the treatment of solid tumors. This delay is also due to the targeting strategy used in the development of BH3 mimetics, which was first successful for the inhibition of Bcl-2/ Bcl-X_L_, while specific and potent inhibitors of Mcl-1 were only developed years later [7, 9, 36]. Genetic evidence supports a more prominent role of Mcl-1 and Bcl-X_L_ in many solid malignancies. The recent development of selective inhibitors of Mcl-1 and Bcl-X_L_ may pave the way for the clinical application of BH3 mimetics in solid tumors, and multiple inhibitors are currently being tested in clinical trials (reviewed in [5]).

However, several obstacles need to be overcome to enable the clinical use of Bcl-X_L_ and Mcl-1 inhibitors, including the on-target toxicity on normal cells, the frequent occurrence of resistance to single-agent treatment with BH3 mimetics, and the potentially limited penetrance of these compounds into the hypoxic areas deep within solid tumors. To tackle some of these challenges several strategies have been suggested that incorporate BH3 mimetics in existing or rationally designed treatment approaches, in particular the combination of BH3 mimetics with chemotherapeutic drugs or other molecularly-targeted agents [37–40].

We and others have previously shown that the combination of BH3 mimetics targeting Mcl-1 and Bcl-X_L_ is highly synergistic in multiple types of solid tumors [11, 41–45]. Here, we confirm this synergism in spheroid cultures of pediatric cancers. However, the combination of BH3 mimetics with each other may be limited by toxic effects on normal cells with a dependency on Bcl-X_L_ (like platelets) and thus narrow the therapeutic window of Bcl-X_L_ targeting BH3 mimetics [16]. While the safety profile of BH3 mimetics targeting Mcl-1 is not yet determined, preclinical studies indicate potential cardiac toxicity, again limiting the dose as well as the type of safely applicable drug combinations [18, 19, 46].

Here, we present a novel therapeutic strategy to apply BH3 mimetics for the treatment of solid tumors. Thereby, we provide the first report showing that BH3 mimetics may synergize with NK cell-based immunotherapy. In a similar approach, it has recently been shown that the BH3 mimetics ABT-737, ABT-199, and S63845 synergized with CAR-T cells to kill malignant B cells [47, 48]. Our study demonstrates that BH3 mimetics may synergize with allogeneic activated NK cells to kill solid tumor spheroids. Since NK cells directly activate apoptosis via several routes in the targeted tumor cells, the combination with BH3 mimetics may serve as a second hit, thus overcoming intrinsic apoptosis resistance in tumor cells and triggering NK cell-mediated killing. Thereby, TRAIL signaling does not seem to contribute to the killing of tumor cells, as blocking of TRAIL using blocking antibodies did not impede NK cell-mediated cytotoxicity. Addition of the broad-range caspase inhibitor zVAD.fmk completely abrogated NK cell-mediated killing, confirming that BH3 mimetics and NK cells cooperate to kill tumor cells by apoptosis.

In a similar approach, we have recently shown that Smac mimetics may synergize with NK cell-mediated killing [49]. Preliminary indications that BH3 mimetics may modulate NK cell attack have been provided by a recent report indicating that the Mcl-1 inhibitor maritoclax may cooperate with NK cells to kill leukemia cells [50]. However, maritoclax may exert additional cellular functions unrelated to the inhibition of Mcl-1 and has been shown to induce cell death also in cells lacking Mcl-1 by induction of reactive oxygen species [51]. Here, the highly selective Mcl-1 inhibitor S63845 has been used, which displays potent on-target activity with very limited unspecific toxicity, indicating that the observed cooperation between S63845 and NK cells is due to selective inhibition of Mcl-1 [9]. In addition, similar effects could be observed by inhibition of Bcl-X_L_ with the selective inhibitor A1331852 [52]. We have previously shown that in RMS, both Mcl-1 and Bcl-X_L_ are highly important for survival, but that neutralization of one of these essential prosurvival proteins only leads to limited induction of apoptosis. Mechanistically, this can be explained by the binding of the proapoptotic BH3-only protein Bim to both Mcl-1 and Bcl-X_L_ and a shift of Bim binding from either to the other upon single inhibition [11]. Our finding that either A1331852 or S63845 may synergize with NK cells indicates that single inhibition of either Bcl-X_L_ or Mcl-1 may be sufficient to lower the apoptotic threshold and facilitate NK cell-mediated killing. Which of these proteins is a better target most likely depends on the cell’s intrinsic repertoire of apoptotic players, and the functional importance of Mcl-1 or Bcl-X_L_ in each cell. Here, the RD-GFP spheroids were more sensitized to NK cell-mediated cytotoxicity by the Mcl-1 inhibitor S63845, while the RH30-GFP cells were sensitized by either Mcl-1 inhibition or the Bcl-X_L_ inhibitor A1331852. Of note, the Bcl-2 inhibitor ABT-199 did not increase NK cell-mediated killing in either cell line, supporting our previous finding that Bcl-2 is a less promising target in these cells [11, 53]. In order to translate this discovery into clinical application, BH3-profiling may be used as a predictive biomarker to investigate each tumor’s dependency on the individual antiapoptotic proteins [54, 55]. This may allow the rational selection of the most promising BH3 mimetic to be included in the NK cell-based immunotherapy.

The successful combination of NK cell immunotherapies with BH3 mimetics relies on two factors. First, NK cell immunotherapy needs to be safe and not deteriorate the toxicity profile of BH3 mimetics. Second, NK cells themselves could be susceptible to BH3 mimetics, thereby impeding NK cell-mediated tumor killing and reducing the clinical efficacy of NK cells. Several reports have described the antiapoptotic Bcl-2 proteins as important survival proteins in NK cells [56–58]. Recently, the genetic inhibition of either Bcl-2 or Mcl-1 in mouse NK cells has been shown to ablate NK cell attack upon bone marrow transplantation. In addition, inhibition of Bcl-2 by ABT-199 has also been shown to induce cell death in human NK cells. However, this was prevented by stimulation with IL-15, suggesting that activation renders NK cells resistant to ABT-199 [59]. Thereby, activation of NK cells and the coinciding increased proliferation may induce Mcl-1 expression as the dominant antiapoptotic Bcl-2 protein in NK cells [56]. Our data provide evidence that activated NK cells are not only resistant to ABT-199, but also to the inhibition of Mcl-1 or Bcl-X_L_, thus supporting a combination therapy of BH3 mimetics with activated NK cells.

In conclusion, our study shows that NK cells may be able to synergize with BH3 mimetics to induce apoptosis particularly in apoptosis-resistant areas within solid tumors. The combination of BH3 mimetics and NK cells is able to induce apoptosis at lower concentrations than either approach alone, and thus may be advantageous in overcoming toxicity of BH3 mimetics while maintaining efficacy.

## Material and methods

### Materials

For induction of apoptosis the BH3 mimetics A1331852, ABT-199 (Selleck Chemicals, Houston, TX) or S63845 (ApexBio, Houston, TX), and human recombinant TRAIL (R&D Systems, Wiesbaden, Germany) were used. To inhibit cell death zVAD.fmk (Bachem, Heidelberg, Germany) or anti-TRAIL antibody (#2E5, Enzo Life Sciences, Lörrach, Germany) were added to the experiments.

### Cell culture

GFP/luciferin expressing RH30 cells were generated as previously described for RD-GFP/Luc cells [32]. For convenience, these cells were labelled as RH30/RD-GFP throughout the text. RH30-GFP was cultured in RPMI-1640 medium (Life Technologies, Eggenstein, Germany). RD-GFP was cultivated in DMEM medium (Life Technologies). Both media were supplemented with 10% fetal calf serum (Biochrom, Berlin, Germany), 1% penicillin/streptavidin, and 1 mM sodium pyruvate (Invitrogen, Karlsruhe, Germany). All cells were authenticated by STR profiling and routinely tested by PCR to exclude mycoplasma contamination.

### Generation of 3D tumor spheroids

Tumor spheroids were generated using 96-well ultra-low attachment (ULA) plates (Corning, NY, USA) as described previously [33]. During assay establishment, various cell numbers (1000–20 000) were seeded in 100 μl cell culture medium per well and centrifuged at 1000x *g*, for 10 min at room temperature. Spheroids were grown for 3 days before treatment with BH3 mimetics.

### NK Isolation, expansion, and labelling

Buffy coats of healthy donors were provided by the DRK Blutspendedienst (Frankfurt, Germany). Peripheral blood mononuclear cells were isolated by density gradient centrifugation using Histopaque-1077 (Sigma-Aldrich, Taufkirchen, Germany) and NK cells were enriched by immunomagnetic negative selection according to manufacturer’s instruction (EasySep Human NK Cell Enrichment Kit, Stem Cell Technologies, Vancouver, Canada) followed by expansion and activation with IL-15 (Peprotech, Rocky Hill, CT) for 15 days (Supplementary Fig. 1).

### Cell death analysis

Viability was assessed in 2D monolayer culture as well as 3D spheroid culture after treatment with indicated concentrations of BH3 mimetics for 48 h using CellTiter-Glo® viability assay (Promega, Mannheim, Germany). Analysis of cell death in spheroids was performed by uptake of 2 μg/ml PI (Sigma-Aldrich) for 30 min in 96 well plates followed by imaging at ImageXpress Micro XLS Widefield Analysis System.

### Statistical analysis

The numbers of independent repetitions (sample size) for each experiment are indicated in the figure legends and were determined by the standard deviation (S.D.). Cell line-based experiments were considered reliable if the S.D. did not exceed 10% within the replicates and repetitions. Bliss Synergy Score was calculated based on the viability data using the synergyfinder web application version 1 (https://synergyfinder.fimm.fi). Statistical significance of NK cell-mediated cytotoxicity in spheroids was investigated in GraphPad Prism7 using repeated measurements and two-way ANOVA followed by Bonferroni’s Multiple Comparison Analysis.

## Supplementary information

Authorship Agreement

Reproducibility Checklist

Supplementary Methods

Supplementary Figure Legends

Supplementary Figures

Video 1

Video 2

Video 3

## Data Availability

All data are available upon request from the corresponding author. Additional methods can be found in the Supplementary data.
