# Supplementary material for: Pediatric multicellular tumor spheroid models illustrate a therapeutic potential by combining BH3 mimetics with Natural Killer (NK) cell-based immunotherapy
Source: Cell Death Discov. 2022 Jan 10;8:11. doi: 10.1038/s41420-021-00812-6 (PMC8748928; doi:10.1038/s41420-021-00812-6)
Supplement: Supplementary file 1 — Authorship Agreement [file 41420_2021_812_MOESM1_ESM.pdf]

Dear Meike,

I agree to the change in authorship.

Viele Grüße,

Carsten

Am 13.12.2021 um 09:42 schrieb Dr. Meike Vogler <m.vogler@kinderkrebsstiftung-frankfurt.de>:

Dear all,

the journal CDDisc would like us all to approve the addition of Sara Wiedemann as additional author to our manuscript. Sara is a new colleague who has helped us with the experiments during the revision. This confirmation is a common formality requried when you add authors during revisions.

Could I please ask you all to reply to this email stating that you agree to the change in authorship as per the journal's instructions below?

"It has come to our attention that your most recent author list differs from the one in your original submission. We find that the following authors have been added since your initial submission: Sara Wiedemann

Please request agreement from all authors including additions and deletions, these can be collected in the following way:

Email your co-authors with the change, and ask them to reply to your email confirming that they agree to these changes. Once you have collected these replies, please combine all of the co-authors' email responses in one document and upload this file to your submission."

Thanks a lot,

Meike

Dr. Meike Vogler

Laborleitung
Institut für Experimentelle Tumorforschung in der Pädiatrie
Goethe-Universität Frankfurt
Im Dr. Petra Joh-Haus der Frankfurter Stiftung für krebskranke Kinder
Komturstraße 3a
60528 Frankfurt a. M.
Tel.: 069 678 665 -74
E-Mail: m.vogler@kinderkrebsstiftung-frankfurt.de
Internet: http://www.kinderkrebsstiftung-frankfurt.de

To: v.saerchen@kinderkrebsstiftung-frankfurt.de
s.shanmugalingam@kinderkrebsstiftung-frankfurt.de
maureenjacob@gmx.net

sarahkehr@gmx.de
LisaMarie.Reindl@kgu.de
Sibylle.Wehner@kgu.de
Victoria.Greze@kgu.de
c.boedicker@kinderkrebsstiftung-frankfurt.de
Katrin.Bankov@kgu.de
Nina.Becker@kgu.de
Till-Martin.Theilen@kgu.de
steffen.gretser@kgu.de
Elise.Gradhand@kgu.de
carsten.kummerow@gmail.com
Evelyn.Ullrich@kgu.de
s.wiedemann@kinderkrebsstiftung-frankfurt.de

Dear Meike,

I agree with the change of authors.

Best,

Cathinka

Original Message processed by david®

CDDISCOVERY-21-2714R1 - Confirmation of authorship December/13 2021, 09:42 AM

Von Dr. Meike Vogler

An (16) Vinzenz Särchen | Senthan Shanmugalingam | maureenjacob@gmx.net | Sarah Kehr | LisaMarie.Reindl@kgu.de | sibylle.wehner@kgu.de | Victoria.Greze@kgu.de | Cathinka Boedicker | Katrin.Bankov@kgu.de | Nina.Becker@kgu.de | Theilen, Till | steffen.gretser@kgu.de | Elise.Gradhand@kgu.de | carsten.kummerow@gmail.com | Evelyn.Ullrich@kgu.de | Sara Wiedemann

Hohe Priorität

Dear all,

the journal CDDisc would like us all to approve the addition of Sara Wiedemann as additional author to our manuscript. Sara is a new colleague who has helped us with the experiments during the revision. This confirmation is a common formality requried when you add authors during revisions.

Could I please ask you all to reply to this email stating that you agree to the change in authorship as per the journal's instructions below?

"It has come to our attention that your most recent author list differs from the one in your original submission. We find that the following authors have been added since your initial submission: Sara Wiedemann

Please request agreement from all authors including additions and deletions, these can be collected in the following way:

Email your co-authors with the change, and ask them to reply to your email confirming that they agree to these changes. Once you have collected these replies, please combine all of the co-authors' email responses in one document and upload this file to your submission."

Thanks a lot,

Meike

Dr. Meike Vogler

Laborleitung
Institut für Experimentelle Tumorforschung in der Pädiatrie
Goethe-Universität Frankfurt
Im Dr. Petra Joh-Haus der Frankfurter Stiftung für krebskranke Kinder
Komturstraße 3a
60528 Frankfurt a. M.
Tel.: 069 678 665 -74

E-Mail: m.vogler@kinderkrebsstiftung-frankfurt.de
Internet: http://www.kinderkrebsstiftung-frankfurt.de

To: v.saerchen@kinderkrebsstiftung-frankfurt.de
s.shanmugalingam@kinderkrebsstiftung-frankfurt.de
maureenjacob@gmx.net
sarahkehr@gmx.de
LisaMarie.Reindl@kgu.de
Sibylle.Wehner@kgu.de
Victoria.Greze@kgu.de
c.boedicker@kinderkrebsstiftung-frankfurt.de
Katrin.Bankov@kgu.de
Nina.Becker@kgu.de
Till-Martin.Theilen@kgu.de
steffen.gretser@kgu.de
Elise.Gradhand@kgu.de
carsten.kummerow@gmail.com
Evelyn.Ullrich@kgu.de
s.wiedemann@kinderkrebsstiftung-frankfurt.de

To whom it may concern
I agree to the change of authorship and thereofre I am happy that Sara Wiedemann is added to our author´s list.
Kind regards
Elise Gradhand

Dr. med. Elise Gradhand, FRCPath (Paeds)

FÄ für Pathologie mit Subspezialisierung in Kinder- und Perinatalpathologie

Geschäftsführende Oberärztin

Sektionsleiterin Kinder- und Perinatalpathologie
Dr. Senckenberg. Institut für Pathologie
Universitätsklinikum Frankfurt/Main
Theodor-Stern-Kai 7
60590 Frankfurt

Telefon direkt: +49 69 6301-85699
Telefon: +049 69 6301-5129 (Sekretariat)
Fax: +49 69 6301-7833 (Sekretariat)
elise.gradhand@kgu.de

Von: Dr. Meike Vogler <m.vogler@kinderkrebsstiftung-frankfurt.de>
Gesendet: Montag, 13. Dezember 2021 09:42
An: Vinzenz Särchen <v.saerchen@kinderkrebsstiftung-frankfurt.de>; Senthan Shanmugalingam <s.shanmugalingam@kinderkrebsstiftung-frankfurt.de>; maureenjacob@gmx.net; Sarah Kehr <sarahkehr@gmx.de>; Reindl, Lisa Marie <LisaMarie.Reindl@kgu.de>; Wehner, Sibylle <Sibylle.Wehner@kgu.de>; Greze, Victoria <Victoria.Greze@kgu.de>; Cathinka Boedicker <c.boedicker@kinderkrebsstiftung-frankfurt.de>; Bankov, Katrin <Katrin.Bankov@kgu.de>; Becker, Nina <Nina.Becker@kgu.de>; Theilen, Till <Till-Martin.Theilen@kgu.de>; Gretser, Steffen <Steffen.Gretser@kgu.de>; Gradhand, Elise Dr. <Elise.Gradhand@kgu.de>; carsten.kummerow@gmail.com; Ullrich, Evelyn Prof. Dr. <Evelyn.Ullrich@kgu.de>; Sara Wiedemann <s.wiedemann@kinderkrebsstiftung-frankfurt.de>
Betreff: CDDISCOVERY-21-2714R1 - Confirmation of authorship
Priorität: Hoch

Dear all,

the journal CDDisc would like us all to approve the addition of Sara Wiedemann as additional author to our manuscript. Sara is a new colleague who has helped us with the experiments during the revision. This confirmation is a common formality requried when you add authors during revisions.

Could I please ask you all to reply to this email stating that you agree to the change in authorship as per the journal's instructions below?

"It has come to our attention that your most recent author list differs from the one in your original submission. We find that the following authors have been added since your initial submission: Sara Wiedemann

Please request agreement from all authors including additions and deletions, these can be collected in the following way:

Email your co-authors with the change, and ask them to reply to your email confirming that they agree to these changes. Once you have collected these replies, please combine all of the co-authors? email responses in one document and upload this file to your submission."

Thanks a lot,

Meike

Dr. Meike Vogler

Laborleitung
Institut für Experimentelle Tumorforschung in der Pädiatrie
Goethe-Universität Frankfurt
Im Dr. Petra Joh-Haus der Frankfurter Stiftung für krebskranke Kinder
Komturstraße 3a
60528 Frankfurt a. M.
Tel.: 069 678 665 -74
E-Mail: m.vogler@kinderkrebsstiftung-frankfurt.de
Internet: https://ddei3-0-ctp.trendmicro.com:443/wis/clicktime/v1/query?url=http%3a%2f%2fwww.kinderkrebsstiftung%2dfrankfurt.de&umid=D9E427C5-D303-0D05-A5D8-02D8D8301319&auth=0add78fbf5bb9f3932276e031687124c15a6990b-2544aa0cbd40e86216140a1096c41935b2e88584

To: v.saerchen@kinderkrebsstiftung-frankfurt.de
s.shanmugalingam@kinderkrebsstiftung-frankfurt.de
maureenjacob@gmx.net
sarahkehr@gmx.de
LisaMarie.Reindl@kgu.de
Sibylle.Wehner@kgu.de
Victoria.Greze@kgu.de
c.boedicker@kinderkrebsstiftung-frankfurt.de
Katrin.Bankov@kgu.de

Nina.Becker@kgu.de
Till-Martin.Theilen@kgu.de
steffen.gretser@kgu.de
Elise.Gradhand@kgu.de
carsten.kummerow@gmail.com
Evelyn.Ullrich@kgu.de
s.wiedemann@kinderkrebsstiftung-frankfurt.de

Dear Meike,
herewith, I agree to the change in authorship as submitted on the revised version.
Best regards,
Evelyn

Univ.-Prof. Dr. med.
Evelyn Ullrich

Universitätsklinikum Frankfurt
Klinik für Kinder- und Jugendmedizin
Experimentelle Immunologie
Johanna Quandt Zentrum
Theodor-Stern-Kai 7 / Haus 32E / R209E
D-60590 Frankfurt

Sekretariat: +49 69 6301-7126
Büro: +49 69 6301-83000
Mobil: +49 151-1719-1871
Intern : 179-1631

Mail: evelyn.ullrich@kgu.de
Web: **Experimental Immunology**

Von: Dr. Meike Vogler <m.vogler@kinderkrebsstiftung-frankfurt.de>
Gesendet: Montag, 13. Dezember 2021 09:42
An: Vinzenz Särchen <v.saerchen@kinderkrebsstiftung-frankfurt.de>; Senthan Shanmugalingam <s.shanmugalingam@kinderkrebsstiftung-frankfurt.de>; maureenjacob@gmx.net; Sarah Kehr <sarahkehr@gmx.de>; Reindl, Lisa Marie <LisaMarie.Reindl@kgu.de>; Wehner, Sibylle <Sibylle.Wehner@kgu.de>; Greze, Victoria <Victoria.Greze@kgu.de>; Cathinka Boedicker <c.boedicker@kinderkrebsstiftung-frankfurt.de>; Bankov, Katrin <Katrin.Bankov@kgu.de>; Becker, Nina <Nina.Becker@kgu.de>; Theilen, Till <Till-Martin.Theilen@kgu.de>; Gretser, Steffen <Steffen.Gretser@kgu.de>; Gradhand, Elise Dr. <Elise.Gradhand@kgu.de>; carsten.kummerow@gmail.com; Ullrich, Evelyn Prof. Dr. <Evelyn.Ullrich@kgu.de>; Sara Wiedemann <s.wiedemann@kinderkrebsstiftung-frankfurt.de>
Betreff: CDDISCOVERY-21-2714R1 - Confirmation of authorship
Priorität: Hoch

Dear all,

the journal CDDisc would like us all to approve the addition of Sara Wiedemann as additional author to our manuscript. Sara is a new colleague who has helped us with the experiments during the revision. This confirmation is a common formality requried when you add authors during revisions.

Could I please ask you all to reply to this email stating that you agree to the change in authorship as per the journal's instructions below?

"It has come to our attention that your most recent author list differs from the one in your original submission. We find that the following authors have been added since your initial submission: Sara Wiedemann

Please request agreement from all authors including additions and deletions, these can be collected in the following way:

Email your co-authors with the change, and ask them to reply to your email confirming that they agree to these changes. Once you have collected these replies, please combine all of the co-authors? email responses in one document and upload this file to your submission."

Thanks a lot,

Meike

Dr. Meike Vogler

Laborleitung
Institut für Experimentelle Tumorforschung in der Pädiatrie
Goethe-Universität Frankfurt
Im Dr. Petra Joh-Haus der Frankfurter Stiftung für krebskranke Kinder
Komturstraße 3a
60528 Frankfurt a. M.
Tel.: 069 678 665 -74
E-Mail: m.vogler@kinderkrebsstiftung-frankfurt.de
Internet: https://ddei3-0-ctp.trendmicro.com:443/wis/clicktime/v1/query?url=http%3a%2f%2fwww.kinderkrebsstiftung%2dfrankfurt.de&umid=D9DFCBDA-D303-0D05-832B-2DB2CDD33201&auth=0add78fbf5bb9f3932276e031687124c15a6990b-fda18de959e82438966c8c8ba26a2319a9f9f536

To: v.saerchen@kinderkrebsstiftung-frankfurt.de
s.shanmugalingam@kinderkrebsstiftung-frankfurt.de
maureenjacob@gmx.net
sarahkehr@gmx.de
LisaMarie.Reindl@kgu.de
Sibylle.Wehner@kgu.de
Victoria.Greze@kgu.de
c.boedicker@kinderkrebsstiftung-frankfurt.de
Katrin.Bankov@kgu.de
Nina.Becker@kgu.de
Till-Martin.Theilen@kgu.de
steffen.gretser@kgu.de
Elise.Gradhand@kgu.de
carsten.kummerow@gmail.com
Evelyn.Ullrich@kgu.de
s.wiedemann@kinderkrebsstiftung-frankfurt.de

To whom it may concern,

I hereby agree **to the change in authorship by adding Sara Wiedemann.**

With kind regards,

Dr. phil. nat. Dipl. Ing.
Katrin Bankov
Leitung Senckenberg Biobank

Universitätsklinikum Frankfurt
Dr. Senckenberg. Institut für Pathologie
Senckenberg BioBank (SBB)
Theodor-Stern-Kai 7
D-60590 Frankfurt
Telefon: +49 69 6301-5387
Fax: +49 69 6301-5456
Mail: katrin.bankov@kgu.de
sbb.pathologie@kgu.de
Web: www.kgu.de

Von: Dr. Meike Vogler [mailto:m.vogler@kinderkrebsstiftung-frankfurt.de]
Gesendet: Montag, 13. Dezember 2021 09:42
An: Vinzenz Särchen <v.saerchen@kinderkrebsstiftung-frankfurt.de>; Senthan Shanmugalingam <s.shanmugalingam@kinderkrebsstiftung-frankfurt.de>; maureenjacob@gmx.net; Sarah Kehr <sarahkehr@gmx.de>; Reindl, Lisa Marie <LisaMarie.Reindl@kgu.de>; Wehner, Sibylle <Sibylle.Wehner@kgu.de>; Greze, Victoria <Victoria.Greze@kgu.de>; Cathinka Boedicker <c.boedicker@kinderkrebsstiftung-frankfurt.de>; Bankov, Katrin <Katrin.Bankov@kgu.de>; Becker, Nina <Nina.Becker@kgu.de>; Theilen, Till <Till-Martin.Theilen@kgu.de>; Gretser, Steffen <Steffen.Gretser@kgu.de>; Gradhand, Elise Dr. <Elise.Gradhand@kgu.de>; carsten.kummerow@gmail.com; Ullrich, Evelyn Prof. Dr. <Evelyn.Ullrich@kgu.de>; Sara Wiedemann <s.wiedemann@kinderkrebsstiftung-frankfurt.de>
Betreff: CDDISCOVERY-21-2714R1 - Confirmation of authorship
Priorität: Hoch

Dear all,

the journal CDDisc would like us all to approve the addition of Sara Wiedemann as additional author to our manuscript. Sara is a new colleague who has helped us with the experiments during the revision. This confirmation is a common formality requried when you add authors during revisions.

Could I please ask you all to reply to this email stating that you agree to the change in authorship as per the journal's instructions below?

"It has come to our attention that your most recent author list differs from the one in your original submission. We find that the following authors have been added since your initial submission: Sara Wiedemann

Please request agreement from all authors including additions and deletions, these can be collected in the following way:

Email your co-authors with the change, and ask them to reply to your email confirming that they agree to these changes. Once you have collected these replies, please combine all of the co-authors? email responses in one document and upload this file to your submission."

Thanks a lot,

Meike

Dr. Meike Vogler

Laborleitung

Institut für Experimentelle Tumorforschung in der Pädiatrie

Goethe-Universität Frankfurt

Im Dr. Petra Joh-Haus der Frankfurter Stiftung für krebskranke Kinder

Komturstraße 3a

60528 Frankfurt a. M.

Tel.: 069 678 665 -74

E-Mail: m.vogler@kinderkrebsstiftung-frankfurt.de

Internet: https://ddei3-0-ctp.trendmicro.com:443/wis/clicktime/v1/query?url=http%3a%2f%2fwww.kinderkrebsstiftung%2dfrankfurt.de&umid=D9DDAE61-D303-0D05-AFC9-4290FE21C90C&auth=0add78fbf5bb9f3932276e031687124c15a6990b-2dc9191b0576fb7aae64cdda90bbac686295284c

To: v.saerchen@kinderkrebsstiftung-frankfurt.de
s.shanmugalingam@kinderkrebsstiftung-frankfurt.de
maureenjacob@gmx.net
sarahkehr@gmx.de
LisaMarie.Reindl@kgu.de
Sibylle.Wehner@kgu.de
Victoria.Greze@kgu.de
c.boedicker@kinderkrebsstiftung-frankfurt.de
Katrin.Bankov@kgu.de

Nina.Becker@kgu.de
Till-Martin.Theilen@kgu.de
steffen.gretser@kgu.de
Elise.Gradhand@kgu.de
carsten.kummerow@gmail.com
Evelyn.Ullrich@kgu.de
s.wiedemann@kinderkrebsstiftung-frankfurt.de

Dear Meike,

I agree to the change in authorship.

Best regards,
Lisa Marie

Von: Dr. Meike Vogler [mailto:m.vogler@kinderkrebsstiftung-frankfurt.de]
Gesendet: Montag, 13. Dezember 2021 09:42
An: Vinzenz Särchen <v.saerchen@kinderkrebsstiftung-frankfurt.de>; Senthan Shanmugalingam <s.shanmugalingam@kinderkrebsstiftung-frankfurt.de>; maureenjacob@gmx.net; Sarah Kehr <sarahkehr@gmx.de>; Reindl, Lisa Marie <LisaMarie.Reindl@kgu.de>; Wehner, Sibylle <Sibylle.Wehner@kgu.de>; Greze, Victoria <Victoria.Greze@kgu.de>; Cathinka Boedicker <c.boedicker@kinderkrebsstiftung-frankfurt.de>; Bankov, Katrin <Katrin.Bankov@kgu.de>; Becker, Nina <Nina.Becker@kgu.de>; Theilen, Till <Till-Martin.Theilen@kgu.de>; Gretser, Steffen <Steffen.Gretser@kgu.de>; Gradhand, Elise Dr. <Elise.Gradhand@kgu.de>; carsten.kummerow@gmail.com; Ullrich, Evelyn Prof. Dr. <Evelyn.Ullrich@kgu.de>; Sara Wiedemann <s.wiedemann@kinderkrebsstiftung-frankfurt.de>
Betreff: CDDISCOVERY-21-2714R1 - Confirmation of authorship
Priorität: Hoch

Dear all,

the journal CDDisc would like us all to approve the addition of Sara Wiedemann as additional author to our manuscript. Sara is a new colleague who has helped us with the experiments during the revision. This confirmation is a common formality requried when you add authors during revisions.

Could I please ask you all to reply to this email stating that you agree to the change in authorship as per the journal's instructions below?

"It has come to our attention that your most recent author list differs from the one in your original submission. We find that the following authors have been added since your initial submission: Sara Wiedemann

Please request agreement from all authors including additions and deletions, these can be collected in the following way:

Email your co-authors with the change, and ask them to reply to your email confirming that they agree to these changes. Once you have collected these replies, please combine all of the co-authors? email responses in one document and upload this file to your submission."

Thanks a lot,

Meike

Dr. Meike Vogler

Laborleitung
Institut für Experimentelle Tumorforschung in der Pädiatrie
Goethe-Universität Frankfurt
Im Dr. Petra Joh-Haus der Frankfurter Stiftung für krebskranke Kinder
Komturstraße 3a
60528 Frankfurt a. M.
Tel.: 069 678 665 -74
E-Mail: m.vogler@kinderkrebsstiftung-frankfurt.de
Internet: https://ddei3-0-ctp.trendmicro.com:443/wis/clicktime/v1/query?url=http%3a%2f%2fwww.kinderkrebsstiftung%2dfrankfurt.de&umid=B6E619E6-D303-0D05-B59E-490EDF553341&auth=0add78fbf5bb9f3932276e031687124c15a6990b-c094fb104e593ad66efc467a07801a28c81e5454

To: v.saerchen@kinderkrebsstiftung-frankfurt.de
s.shanmugalingam@kinderkrebsstiftung-frankfurt.de
maureenjacob@gmx.net
sarahkehr@gmx.de
LisaMarie.Reindl@kgu.de
Sibylle.Wehner@kgu.de
Victoria.Greze@kgu.de
c.boedicker@kinderkrebsstiftung-frankfurt.de
Katrin.Bankov@kgu.de
Nina.Becker@kgu.de
Till-Martin.Theilen@kgu.de
steffen.gretser@kgu.de
Elise.Gradhand@kgu.de
carsten.kummerow@gmail.com
Evelyn.Ullrich@kgu.de
s.wiedemann@kinderkrebsstiftung-frankfurt.de

Dear Meike,

I agree with the change of authorship that Sara Wiedemann is added to the author list.

Best,
Maureen Jacob

Am 13.12.2021 um 09:42 schrieb Dr. Meike Vogler <m.vogler@kinderkrebsstiftung-frankfurt.de>:

Dear all,

the journal CDDisc would like us all to approve the addition of Sara Wiedemann as additional author to our manuscript. Sara is a new colleague who has helped us with the experiments during the revision. This confirmation is a common formality requried when you add authors during revisions.

Could I please ask you all to reply to this email stating that you agree to the change in authorship as per the journal's instructions below?

"It has come to our attention that your most recent author list differs from the one in your original submission. We find that the following authors have been added since your initial submission: Sara Wiedemann

Please request agreement from all authors including additions and deletions, these can be collected in the following way:

Email your co-authors with the change, and ask them to reply to your email confirming that they agree to these changes. Once you have collected these replies, please combine all of the co-authors' email responses in one document and upload this file to your submission."

Thanks a lot,

Meike

Dr. Meike Vogler

Laborleitung
Institut für Experimentelle Tumorforschung in der Pädiatrie
Goethe-Universität Frankfurt
Im Dr. Petra Joh-Haus der Frankfurter Stiftung für krebskranke Kinder
Komturstraße 3a
60528 Frankfurt a. M.
Tel.: 069 678 665 -74
E-Mail: m.vogler@kinderkrebsstiftung-frankfurt.de
Internet: http://www.kinderkrebsstiftung-frankfurt.de

To: v.saerchen@kinderkrebsstiftung-frankfurt.de
s.shanmugalingam@kinderkrebsstiftung-frankfurt.de

maureenjacob@gmx.net
sarahkehr@gmx.de
LisaMarie.Reindl@kgu.de
Sibylle.Wehner@kgu.de
Victoria.Greze@kgu.de
c.boedicker@kinderkrebsstiftung-frankfurt.de
Katrin.Bankov@kgu.de
Nina.Becker@kgu.de
Till-Martin.Theilen@kgu.de
steffen.gretser@kgu.de
Elise.Gradhand@kgu.de
carsten.kummerow@gmail.com
Evelyn.Ullrich@kgu.de
s.wiedemann@kinderkrebsstiftung-frankfurt.de

Dear Meike,
I agree to the change in authorship as per the journal´s instructions below.

Best regards, Nina Becker

Von: Dr. Meike Vogler <m.vogler@kinderkrebsstiftung-frankfurt.de>
Gesendet: Montag, 13. Dezember 2021 09:42
An: Vinzenz Särchen <v.saerchen@kinderkrebsstiftung-frankfurt.de>; Senthan Shanmugalingam <s.shanmugalingam@kinderkrebsstiftung-frankfurt.de>; maureenjacob@gmx.net; Sarah Kehr <sarahkehr@gmx.de>; Reindl, Lisa Marie <LisaMarie.Reindl@kgu.de>; Wehner, Sibylle <Sibylle.Wehner@kgu.de>; Greze, Victoria <Victoria.Greze@kgu.de>; Cathinka Boedicker <c.boedicker@kinderkrebsstiftung-frankfurt.de>; Bankov, Katrin <Katrin.Bankov@kgu.de>; Becker, Nina <Nina.Becker@kgu.de>; Theilen, Till <Till-Martin.Theilen@kgu.de>; Gretser, Steffen <Steffen.Gretser@kgu.de>; Gradhand, Elise Dr. <Elise.Gradhand@kgu.de>; carsten.kummerow@gmail.com; Ullrich, Evelyn Prof. Dr. <Evelyn.Ullrich@kgu.de>; Sara Wiedemann <s.wiedemann@kinderkrebsstiftung-frankfurt.de>
Betreff: CDDISCOVERY-21-2714R1 - Confirmation of authorship
Priorität: Hoch

Dear all,

the journal CDDisc would like us all to approve the addition of Sara Wiedemann as additional author to our manuscript. Sara is a new colleague who has helped us with the experiments during the revision. This confirmation is a common formality requried when you add authors during revisions.

Could I please ask you all to reply to this email stating that you agree to the change in authorship as per the journal's instructions below?

"It has come to our attention that your most recent author list differs from the one in your original submission. We find that the following authors have been added since your initial submission: Sara Wiedemann

Please request agreement from all authors including additions and deletions, these can be collected in the following way:

Email your co-authors with the change, and ask them to reply to your email confirming that they agree to these changes. Once you have collected these replies, please combine all of the co-authors’ email responses in one document and upload this file to your submission."

Thanks a lot,

Meike

Dr. Meike Vogler

Laborleitung
Institut für Experimentelle Tumorforschung in der Pädiatrie
Goethe-Universität Frankfurt
Im Dr. Petra Joh-Haus der Frankfurter Stiftung für krebskranke Kinder
Komturstraße 3a
60528 Frankfurt a. M.
Tel.: 069 678 665 -74
E-Mail: m.vogler@kinderkrebsstiftung-frankfurt.de
Internet: http://www.kinderkrebsstiftung-frankfurt.de

To: v.saerchen@kinderkrebsstiftung-frankfurt.de
s.shanmugalingam@kinderkrebsstiftung-frankfurt.de
maureenjacob@gmx.net
sarahkehr@gmx.de
LisaMarie.Reindl@kgu.de
Sibylle.Wehner@kgu.de
Victoria.Greze@kgu.de
c.boedicker@kinderkrebsstiftung-frankfurt.de
Katrin.Bankov@kgu.de
Nina.Becker@kgu.de
Till-Martin.Theilen@kgu.de
steffen.gretser@kgu.de
Elise.Gradhand@kgu.de
carsten.kummerow@gmail.com
Evelyn.Ullrich@kgu.de
s.wiedemann@kinderkrebsstiftung-frankfurt.de

Dear Meike,

I agree to the change in authorship.

Best regards,

Sara

Original Message processed by david®

CDDISCOVERY-21-2714R1 - Confirmation of authorship 13. Dezember 2021, 09:42 Uhr

Von Dr. Meike Vogler

An (16) Vinzenz Särchen | Senthan Shanmugalingam | maureenjacob@gmx.net | Sarah Kehr | LisaMarie.Reindl@kgu.de | sibylle.wehner@kgu.de | Victoria.Greze@kgu.de | Cathinka Boedicker | Katrin.Bankov@kgu.de | Nina.Becker@kgu.de | Theilen, Till | steffen.gretser@kgu.de | Elise.Gradhand@kgu.de | carsten.kummerow@gmail.com | Evelyn.Ullrich@kgu.de | Sara Wiedemann

Hohe Priorität

Dear all,

the journal CDDisc would like us all to approve the addition of Sara Wiedemann as additional author to our manuscript. Sara is a new colleague who has helped us with the experiments during the revision. This confirmation is a common formality requried when you add authors during revisions.

Could I please ask you all to reply to this email stating that you agree to the change in authorship as per the journal's instructions below?

"It has come to our attention that your most recent author list differs from the one in your original submission. We find that the following authors have been added since your initial submission: Sara Wiedemann

Please request agreement from all authors including additions and deletions, these can be collected in the following way:

Email your co-authors with the change, and ask them to reply to your email confirming that they agree to these changes. Once you have collected these replies, please combine all of the co-authors’ email responses in one document and upload this file to your submission."

Thanks a lot,

Meike

Dr. Meike Vogler

Laborleitung
Institut für Experimentelle Tumorforschung in der Pädiatrie
Goethe-Universität Frankfurt
Im Dr. Petra Joh-Haus der Frankfurter Stiftung für krebskranke Kinder
Komturstraße 3a
60528 Frankfurt a. M.
Tel.: 069 678 665 -74

E-Mail: m.vogler@kinderkrebsstiftung-frankfurt.de
Internet: http://www.kinderkrebsstiftung-frankfurt.de

```
To: v.saerchen@kinderkrebsstiftung-frankfurt.de
    s.shanmugalingam@kinderkrebsstiftung-frankfurt.de
    maureenjacob@gmx.net
    sarahkehr@gmx.de
    LisaMarie.Reindl@kgu.de
    Sibylle.Wehner@kgu.de
    Victoria.Greze@kgu.de
    c.boedicker@kinderkrebsstiftung-frankfurt.de
    Katrin.Bankov@kgu.de
    Nina.Becker@kgu.de
    Till-Martin.Theilen@kgu.de
    steffen.gretser@kgu.de
    Elise.Gradhand@kgu.de
    carsten.kummerow@gmail.com
    Evelyn.Ullrich@kgu.de
    s.wiedemann@kinderkrebsstiftung-frankfurt.de
```

Dear Meike,

Thank you for your E-Mail.
I agree to the addition of Sara Wiedemann as an author.

Sarah

> **Gesendet:** Montag, 13. Dezember 2021 um 09:42 Uhr
> **Von:** "Dr. Meike Vogler" <m.vogler@kinderkrebsstiftung-frankfurt.de>
> **An:** "Vinzenz Särchen" <v.saerchen@kinderkrebsstiftung-frankfurt.de>, "Senthan Shanmugalingam" <s.shanmugalingam@kinderkrebsstiftung-frankfurt.de>, maureenjacob@gmx.net, "Sarah Kehr" <sarahkehr@gmx.de>, LisaMarie.Reindl@kgu.de, "sibylle.wehner@kgu.de" <Sibylle.Wehner@kgu.de>, Victoria.Greze@kgu.de, "Cathinka Boedicker" <c.boedicker@kinderkrebsstiftung-frankfurt.de>, Katrin.Bankov@kgu.de, Nina.Becker@kgu.de, "Theilen, Till" <Till-Martin.Theilen@kgu.de>, steffen.gretser@kgu.de, Elise.Gradhand@kgu.de, carsten.kummerow@gmail.com, Evelyn.Ullrich@kgu.de, "Sara Wiedemann" <s.wiedemann@kinderkrebsstiftung-frankfurt.de>
> **Betreff:** CDDISCOVERY-21-2714R1 - Confirmation of authorship
>
> Dear all,
>
> the journal CDDisc would like us all to approve the addition of Sara Wiedemann as additional author to our manuscript. Sara is a new colleague who has helped us with the experiments during the revision. This confirmation is a common formality requried when you add authors during revisions.
>
> **Could I please ask you all to reply to this email stating that you agree to the change in authorship as per the journal's instructions below?**
>
> "It has come to our attention that your most recent author list differs from the one in your original submission. We find that the following authors have been added since your initial submission: Sara Wiedemann
>
> Please request agreement from all authors including additions and deletions, these can be collected in the following way:
>
> Email your co-authors with the change, and ask them to reply to your email confirming that they agree to these changes. Once you have collected these replies, please combine all of the co-authors' email responses in one document and upload this file to your submission."
>
> Thanks a lot,
>
> Meike
>
> ---
>
> Dr. Meike Vogler
>
> Laborleitung
> Institut für Experimentelle Tumorforschung in der Pädiatrie
> Goethe-Universität Frankfurt
> Im Dr. Petra Joh-Haus der Frankfurter Stiftung für krebskranke Kinder
> Komturstraße 3a
> 60528 Frankfurt a. M.

Tel.: 069 678 665 -74
E-Mail: m.vogler@kinderkrebsstiftung-frankfurt.de
Internet: http://www.kinderkrebsstiftung-frankfurt.de

To: v.saerchen@kinderkrebsstiftung-frankfurt.de
s.shanmugalingam@kinderkrebsstiftung-frankfurt.de
maureenjacob@gmx.net
sarahkehr@gmx.de
LisaMarie.Reindl@kgu.de
Sibylle.Wehner@kgu.de
Victoria.Greze@kgu.de
c.boedicker@kinderkrebsstiftung-frankfurt.de
Katrin.Bankov@kgu.de
Nina.Becker@kgu.de
Till-Martin.Theilen@kgu.de
steffen.gretser@kgu.de
Elise.Gradhand@kgu.de
carsten.kummerow@gmail.com
Evelyn.Ullrich@kgu.de
s.wiedemann@kinderkrebsstiftung-frankfurt.de

Hi Meike,

I agree to the change in authorship as per the journal's instructions below in the formerly sent e-mail.

Senthan

Am 13.12.2021 09:42 schrieb "Dr. Meike Vogler" <m.vogler@kinderkrebsstiftung-frankfurt.de>:

> Dear all,
>
> the journal CDDisc would like us all to approve the addition of Sara Wiedemann as additional author to our manuscript. Sara is a new colleague who has helped us with the experiments during the revision. This confirmation is a common formality requried when you add authors during revisions.
>
> **Could I please ask you all to reply to this email stating that you agree to the change in authorship as per the journal's instructions below?**
>
> "It has come to our attention that your most recent author list differs from the one in your original submission. We find that the following authors have been added since your initial submission: Sara Wiedemann
>
> Please request agreement from all authors including additions and deletions, these can be collected in the following way:
>
> Email your co-authors with the change, and ask them to reply to your email confirming that they agree to these changes. Once you have collected these replies, please combine all of the co-authors' email responses in one document and upload this file to your submission."
>
> Thanks a lot,
>
> Meike
>
> ---
>
> Dr. Meike Vogler
>
> Laborleitung
> Institut für Experimentelle Tumorforschung in der Pädiatrie
> Goethe-Universität Frankfurt
> Im Dr. Petra Joh-Haus der Frankfurter Stiftung für krebskranke Kinder
> Komturstraße 3a
> 60528 Frankfurt a. M.
> Tel.: 069 678 665 -74
> E-Mail: m.vogler@kinderkrebsstiftung-frankfurt.de
> Internet: http://www.kinderkrebsstiftung-frankfurt.de
>
> To: v.saerchen@kinderkrebsstiftung-frankfurt.de
> s.shanmugalingam@kinderkrebsstiftung-frankfurt.de
> maureenjacob@gmx.net
> sarahkehr@gmx.de
> LisaMarie.Reindl@kgu.de
> Sibylle.Wehner@kgu.de
> Victoria.Greze@kgu.de
> c.boedicker@kinderkrebsstiftung-frankfurt.de
> Katrin.Bankov@kgu.de
> Nina.Becker@kgu.de
> Till-Martin.Theilen@kgu.de

steffen.gretser@kgu.de
Elise.Gradhand@kgu.de
carsten.kummerow@gmail.com
Evelyn.Ullrich@kgu.de
s.wiedemann@kinderkrebsstiftung-frankfurt.de

Dear Meike,
I agree to the change in authorship as per the journal´s instructions below.

Best regards, Sibylle Wehner

Sibylle Wehner
Lab Special Pediatric Hematology

University Hospital Frankfurt
Clinic for Pediatrics and Young Adults (KKJM)
Pediatrics Hematology, Oncology, Hemostaseology
Stem Cell Transplantation and Immunology
Theodor-Stern-Kai 7
D-60590 Frankfurt
Germany

Mobile: +49 15117190251
Mobile intern : 170679
Fax: +49 69 6301-83338
Mail: sibylle.wehner@kgu.de
Web: www.kgu.de

Von: Dr. Meike Vogler <m.vogler@kinderkrebsstiftung-frankfurt.de>
Gesendet: Montag, 13. Dezember 2021 09:42
An: Vinzenz Särchen <v.saerchen@kinderkrebsstiftung-frankfurt.de>; Senthan Shanmugalingam <s.shanmugalingam@kinderkrebsstiftung-frankfurt.de>; maureenjacob@gmx.net; Sarah Kehr <sarahkehr@gmx.de>; Reindl, Lisa Marie <LisaMarie.Reindl@kgu.de>; Wehner, Sibylle <Sibylle.Wehner@kgu.de>; Greze, Victoria <Victoria.Greze@kgu.de>; Cathinka Boedicker <c.boedicker@kinderkrebsstiftung-frankfurt.de>; Bankov, Katrin <Katrin.Bankov@kgu.de>; Becker, Nina <Nina.Becker@kgu.de>; Theilen, Till <Till-Martin.Theilen@kgu.de>; Gretser, Steffen <Steffen.Gretser@kgu.de>; Gradhand, Elise Dr. <Elise.Gradhand@kgu.de>; carsten.kummerow@gmail.com; Ullrich, Evelyn Prof. Dr. <Evelyn.Ullrich@kgu.de>; Sara Wiedemann <s.wiedemann@kinderkrebsstiftung-frankfurt.de>
Betreff: CDDISCOVERY-21-2714R1 - Confirmation of authorship
Priorität: Hoch

Dear all,

the journal CDDisc would like us all to approve the addition of Sara Wiedemann as additional author to our manuscript. Sara is a new colleague who has helped us with the experiments during the revision. This confirmation is a common formality requried when you add authors during revisions.

Could I please ask you all to reply to this email stating that you agree to the change in authorship as per the journal's instructions below?

"It has come to our attention that your most recent author list differs from the one in your original submission. We find that the following authors have been added since your initial submission: Sara Wiedemann

Please request agreement from all authors including additions and deletions, these can be collected in the following way:

Email your co-authors with the change, and ask them to reply to your email confirming that they agree to these changes. Once you have collected these replies, please combine all of the co-authors' email responses in one document and upload this file to your submission."

Thanks a lot,

Meike

Dr. Meike Vogler

Laborleitung
Institut für Experimentelle Tumorforschung in der Pädiatrie
Goethe-Universität Frankfurt
Im Dr. Petra Joh-Haus der Frankfurter Stiftung für krebskranke Kinder
Komturstraße 3a
60528 Frankfurt a. M.
Tel.: 069 678 665 -74
E-Mail: m.vogler@kinderkrebsstiftung-frankfurt.de
Internet: http://www.kinderkrebsstiftung-frankfurt.de

To: v.saerchen@kinderkrebsstiftung-frankfurt.de
s.shanmugalingam@kinderkrebsstiftung-frankfurt.de
maureenjacob@gmx.net
sarahkehr@gmx.de
LisaMarie.Reindl@kgu.de
Sibylle.Wehner@kgu.de
Victoria.Greze@kgu.de
c.boedicker@kinderkrebsstiftung-frankfurt.de

Katrin.Bankov@kgu.de
Nina.Becker@kgu.de
Till-Martin.Theilen@kgu.de
steffen.gretser@kgu.de
Elise.Gradhand@kgu.de
carsten.kummerow@gmail.com
Evelyn.Ullrich@kgu.de
s.wiedemann@kinderkrebsstiftung-frankfurt.de

Dear Meike,

I fully agree to the change in authorship and the inclusion of Sara Wiedemann as an additional author.

Sincerely,

Steffen

Von: Dr. Meike Vogler <m.vogler@kinderkrebsstiftung-frankfurt.de>
Gesendet: Montag, 13. Dezember 2021 09:42
An: Vinzenz Särchen <v.saerchen@kinderkrebsstiftung-frankfurt.de>; Senthan Shanmugalingam <s.shanmugalingam@kinderkrebsstiftung-frankfurt.de>; maureenjacob@gmx.net; Sarah Kehr <sarahkehr@gmx.de>; Reindl, Lisa Marie <LisaMarie.Reindl@kgu.de>; Wehner, Sibylle <Sibylle.Wehner@kgu.de>; Greze, Victoria <Victoria.Greze@kgu.de>; Cathinka Boedicker <c.boedicker@kinderkrebsstiftung-frankfurt.de>; Bankov, Katrin <Katrin.Bankov@kgu.de>; Becker, Nina <Nina.Becker@kgu.de>; Theilen, Till <Till-Martin.Theilen@kgu.de>; Gretser, Steffen <Steffen.Gretser@kgu.de>; Gradhand, Elise Dr. <Elise.Gradhand@kgu.de>; carsten.kummerow@gmail.com; Ullrich, Evelyn Prof. Dr. <Evelyn.Ullrich@kgu.de>; Sara Wiedemann <s.wiedemann@kinderkrebsstiftung-frankfurt.de>
Betreff: CDDISCOVERY-21-2714R1 - Confirmation of authorship
Priorität: Hoch

Dear all,

the journal CDDisc would like us all to approve the addition of Sara Wiedemann as additional author to our manuscript. Sara is a new colleague who has helped us with the experiments during the revision. This confirmation is a common formality requried when you add authors during revisions.

Could I please ask you all to reply to this email stating that you agree to the change in authorship as per the journal's instructions below?

"It has come to our attention that your most recent author list differs from the one in your original submission. We find that the following authors have been added since your initial submission: Sara Wiedemann

Please request agreement from all authors including additions and deletions, these can be collected in the following way:

Email your co-authors with the change, and ask them to reply to your email confirming that they agree to these changes. Once you have collected these replies, please combine all of the co-authors’ email responses in one document and upload this file to your submission."

Thanks a lot,

Meike

Dr. Meike Vogler

Laborleitung
Institut für Experimentelle Tumorforschung in der Pädiatrie
Goethe-Universität Frankfurt
Im Dr. Petra Joh-Haus der Frankfurter Stiftung für krebskranke Kinder
Komturstraße 3a
60528 Frankfurt a. M.
Tel.: 069 678 665 -74
E-Mail: m.vogler@kinderkrebsstiftung-frankfurt.de
Internet: http://www.kinderkrebsstiftung-frankfurt.de

To: v.saerchen@kinderkrebsstiftung-frankfurt.de
s.shanmugalingam@kinderkrebsstiftung-frankfurt.de
maureenjacob@gmx.net
sarahkehr@gmx.de
LisaMarie.Reindl@kgu.de
Sibylle.Wehner@kgu.de
Victoria.Greze@kgu.de
c.boedicker@kinderkrebsstiftung-frankfurt.de
Katrin.Bankov@kgu.de
Nina.Becker@kgu.de
Till-Martin.Theilen@kgu.de
steffen.gretser@kgu.de
Elise.Gradhand@kgu.de
carsten.kummerow@gmail.com
Evelyn.Ullrich@kgu.de
s.wiedemann@kinderkrebsstiftung-frankfurt.de

Dear Meike,
I agree with adding Sara Wiedemann to the author list.
Thank you.
Till-Martin Theilen

Am 13.12.2021 um 09:42 schrieb Dr. Meike Vogler <m.vogler@kinderkrebsstiftung-frankfurt.de>:

Dear all,

the journal CDDisc would like us all to approve the addition of Sara Wiedemann as additional author to our manuscript. Sara is a new colleague who has helped us with the experiments during the revision. This confirmation is a common formality requried when you add authors during revisions.

Could I please ask you all to reply to this email stating that you agree to the change in authorship as per the journal's instructions below?

"It has come to our attention that your most recent author list differs from the one in your original submission. We find that the following authors have been added since your initial submission: Sara Wiedemann

Please request agreement from all authors including additions and deletions, these can be collected in the following way:

Email your co-authors with the change, and ask them to reply to your email confirming that they agree to these changes. Once you have collected these replies, please combine all of the co-authors? email responses in one document and upload this file to your submission."

Thanks a lot,

Meike

Dr. Meike Vogler

Laborleitung
Institut für Experimentelle Tumorforschung in der Pädiatrie
Goethe-Universität Frankfurt
Im Dr. Petra Joh-Haus der Frankfurter Stiftung für krebskranke Kinder
Komturstraße 3a
60528 Frankfurt a. M.
Tel.: 069 678 665 -74
E-Mail: m.vogler@kinderkrebsstiftung-frankfurt.de
Internet: https://ddei3-0-ctp.trendmicro.com:443/wis/clicktime/v1/query?url=http%3a%2f%2fwww.kinderkrebsstiftung%2dfrankfurt.de&umid=D9E23912-D303-0D05-8FA8-3C69B4C3FBCC&auth=0add78fbf5bb9f3932276e031687124c15a6990b-a67a0f9dec78df500f0299394bddec1abef64c30

To: v.saerchen@kinderkrebsstiftung-frankfurt.de
s.shanmugalingam@kinderkrebsstiftung-frankfurt.de
maureenjacob@gmx.net
sarahkehr@gmx.de
LisaMarie.Reindl@kgu.de
Sibylle.Wehner@kgu.de

Victoria.Greze@kgu.de
c.boedicker@kinderkrebsstiftung-frankfurt.de
Katrin.Bankov@kgu.de
Nina.Becker@kgu.de
Till-Martin.Theilen@kgu.de
steffen.gretser@kgu.de
Elise.Gradhand@kgu.de
carsten.kummerow@gmail.com
Evelyn.Ullrich@kgu.de
s.wiedemann@kinderkrebsstiftung-frankfurt.de

Dear Meike,

I agree to these changes.

Victoria Grèze.

Von: Dr. Meike Vogler <m.vogler@kinderkrebsstiftung-frankfurt.de>
Gesendet: Montag, 13. Dezember 2021 09:42:25
An: Vinzenz Särchen; Senthan Shanmugalingam; maureenjacob@gmx.net; Sarah Kehr; Reindl, Lisa Marie; Wehner, Sibylle; Greze, Victoria; Cathinka Boedicker; Bankov, Katrin; Becker, Nina; Theilen, Till; Gretser, Steffen; Gradhand, Elise Dr.; carsten.kummerow@gmail.com; Ullrich, Evelyn Prof. Dr.; Sara Wiedemann
Betreff: CDDISCOVERY-21-2714R1 - Confirmation of authorship

Dear all,

the journal CDDisc would like us all to approve the addition of Sara Wiedemann as additional author to our manuscript. Sara is a new colleague who has helped us with the experiments during the revision. This confirmation is a common formality requried when you add authors during revisions.

Could I please ask you all to reply to this email stating that you agree to the change in authorship as per the journal's instructions below?

"It has come to our attention that your most recent author list differs from the one in your original submission. We find that the following authors have been added since your initial submission: Sara Wiedemann

Please request agreement from all authors including additions and deletions, these can be collected in the following way:

Email your co-authors with the change, and ask them to reply to your email confirming that they agree to these changes. Once you have collected these replies, please combine all of the co-authors' email responses in one document and upload this file to your submission."

Thanks a lot,

Meike

Dr. Meike Vogler

Laborleitung
Institut für Experimentelle Tumorforschung in der Pädiatrie
Goethe-Universität Frankfurt
Im Dr. Petra Joh-Haus der Frankfurter Stiftung für krebskranke Kinder
Komturstraße 3a
60528 Frankfurt a. M.
Tel.: 069 678 665 -74
E-Mail: m.vogler@kinderkrebsstiftung-frankfurt.de
Internet: http://www.kinderkrebsstiftung-frankfurt.de

To: v.saerchen@kinderkrebsstiftung-frankfurt.de
s.shanmugalingam@kinderkrebsstiftung-frankfurt.de
maureenjacob@gmx.net

sarahkehr@gmx.de
LisaMarie.Reindl@kgu.de
Sibylle.Wehner@kgu.de
Victoria.Greze@kgu.de
c.boedicker@kinderkrebsstiftung-frankfurt.de
Katrin.Bankov@kgu.de
Nina.Becker@kgu.de
Till-Martin.Theilen@kgu.de
steffen.gretser@kgu.de
Elise.Gradhand@kgu.de
carsten.kummerow@gmail.com
Evelyn.Ullrich@kgu.de
s.wiedemann@kinderkrebsstiftung-frankfurt.de

I, Vinzenz Särchen, agree with the addition of Sara Wiedemann as a co-author to our manuscript (CDDISCOVERY-21-2714R1).

Original Message processed by david®

CDDISCOVERY-21-2714R1 - Confirmation of authorship 13. Dezember 2021, 09:42 Uhr

Von Dr. Meike Vogler

An (16) Vinzenz Särchen | Senthan Shanmugalingam | maureenjacob@gmx.net | Sarah Kehr | LisaMarie.Reindl@kgu.de | sibylle.wehner@kgu.de | Victoria.Greze@kgu.de | Cathinka Boedicker | Katrin.Bankov@kgu.de | Nina.Becker@kgu.de | Theilen, Till | steffen.gretser@kgu.de | Elise.Gradhand@kgu.de | carsten.kummerow@gmail.com | Evelyn.Ullrich@kgu.de | Sara Wiedemann

Hohe Priorität

Dear all,

the journal CDDisc would like us all to approve the addition of Sara Wiedemann as additional author to our manuscript. Sara is a new colleague who has helped us with the experiments during the revision. This confirmation is a common formality requried when you add authors during revisions.

Could I please ask you all to reply to this email stating that you agree to the change in authorship as per the journal's instructions below?

"It has come to our attention that your most recent author list differs from the one in your original submission. We find that the following authors have been added since your initial submission: Sara Wiedemann

Please request agreement from all authors including additions and deletions, these can be collected in the following way:

Email your co-authors with the change, and ask them to reply to your email confirming that they agree to these changes. Once you have collected these replies, please combine all of the co-authors' email responses in one document and upload this file to your submission."

Thanks a lot,

Meike

Dr. Meike Vogler

Laborleitung
Institut für Experimentelle Tumorforschung in der Pädiatrie
Goethe-Universität Frankfurt
Im Dr. Petra Joh-Haus der Frankfurter Stiftung für krebskranke Kinder
Komturstraße 3a
60528 Frankfurt a. M.
Tel.: 069 678 665 -74
E-Mail: m.vogler@kinderkrebsstiftung-frankfurt.de
Internet: http://www.kinderkrebsstiftung-frankfurt.de

To: v.saerchen@kinderkrebsstiftung-frankfurt.de
s.shanmugalingam@kinderkrebsstiftung-frankfurt.de
maureenjacob@gmx.net
sarahkehr@gmx.de
LisaMarie.Reindl@kgu.de
Sibylle.Wehner@kgu.de
Victoria.Greze@kgu.de
c.boedicker@kinderkrebsstiftung-frankfurt.de
Katrin.Bankov@kgu.de
Nina.Becker@kgu.de
Till-Martin.Theilen@kgu.de
steffen.gretser@kgu.de
Elise.Gradhand@kgu.de
carsten.kummerow@gmail.com
Evelyn.Ullrich@kgu.de
s.wiedemann@kinderkrebsstiftung-frankfurt.de
