# Supplementary material for: Pediatric multicellular tumor spheroid models illustrate a therapeutic potential by combining BH3 mimetics with Natural Killer (NK) cell-based immunotherapy
Source: Cell Death Discov. 2022 Jan 10;8:11. doi: 10.1038/s41420-021-00812-6 (PMC8748928; doi:10.1038/s41420-021-00812-6)
Supplement: Supplementary file 3 — Supplementary Methods [file 41420_2021_812_MOESM3_ESM.docx]

**Supplementary Methods**

**Additional Cell Culture**

GFP/luciferin expressing RH30, SK-N-AS and UKF-NB3 cells were generated as previously described for RD-GFP/Luc cells (30). For convenience, these cells were labelled as RH30/RD-GFP not RH30/RD-GFP/Luc throughout the text. RH30-GFP and T174 cells were cultured in RPMI-1640 medium (Life Technologies, Eggenstein, Germany). Kym-1 cells were obtained at JCRB Cell Bank (Japan) and cultured in RPMI-1640 medium. TE381.T and SK-N-AS cells were obtained at the American Type Culture Collection (Manassas, VA). U2OS and MG63 cells were kindly provided by M. Nathrath (München, Germany). RD-GFP, TE381.T, U2OS, HS5 and MG63 were cultivated in DMEM medium (Life Technologies). SK-N-AS and UKF-NB3 in IMDM medium (Life Technologies). SK-N-AS and UKF-NB3 in IMDM medium (Life Technologies). Medium for Kym-1 cells was supplemented with 1% HEPES (Life Technologies). Tumor samples used in this study were provided by the University Cancer Center Frankfurt (UCT). Written informed consent was obtained for all patients and the study was approved by the institutional Review Board of the UCT and the Ethical Committee at the University Hospital Frankfurt (SPO-04-2015).

**Immunohistochemistry and imaging**

GFP expressing 3D tumor spheroids were imaged by an ImageXpress Micro XLS Widefield Analysis System (Molecular Devices, Sunnydale, CA). For each spheroid and each channel (transmitted light, GFP/FITC, propidium iodide (PI)/TRITC, CellTrace™ Violet/DAPI, CellTrace™ far red/Cy5) a series of z-stacks, composed of 25 layers, was acquired. To minimize data storage one best focus projection image was saved into the database for each channel per spheroid. Image analysis was performed using a custom module within MetaXpress (Version 6.5.4.532, Molecular Devices, Sunnydale, CA) and FIJI (ImageJ version 1.53c) (32).

For light sheet microscopy tumor spheroids were embedded in a 4 mg/ml collagen matrix for 1h at 37°C, 5% CO2 and imaged on a Zeiss Lightsheet Z.1 microscope with a Plan-Apo 20x/1.0 objective. A 488 nm laser line was used to excite GFP and PI and z-stacks of fluorescence images were acquired with two PCO.edge cameras using a LP 560 nm beam splitter and a BP 505-530 nm and a LP 585 nm emission filter, respectively. Maximum intensity projections were calculated using ZEN software.

For immunohistochemistry, spheroids were generated using 100 000 cells per well for 3 days before treatment with A1331852 (0.25 μM) and S63845 (0.3 μM) for 6 hours. Treated spheroids were fixed using 10% neutrally buffered formalin for 10 min. Dehydration and paraffin infiltration followed the fully automated routine workflow of the Dr. Senckenberg Institute of Pathology, University Hospital Frankfurt, by the device ASP 6025 (Leica, Wetzlar, Germany). By this workflow, spheroids can be preserved and processed into ultra-thin sections. Immunohistochemistry was performed on ultra-thin sections using the DAKO FLEX-Envision Kit (Agilent, Santa Clara, CA, US) and the fully automated DAKO Omnis staining system (Agilent) according to manufacturer's instruction. Ki-67 primary antibody (Clone MIB-1, GA626, ready to use dilution), was used for immunohistochemical epitope staining applied for 20 min after heat-induced epitope retrieval in pH9 at 97°C. Epitope visualization was done by DAKO EnVision™ FLEX DAB+ Substrate Chromogen System (Agilent) resulting in brown nuclear signal. Nuclear counterstain was done using hematoxylin solution.

Caspase activity of BH3 mimetic pre-treated wild type RMS spheroids (no GFP expression) was measured after 24h after addition of NK cells. CellEvent™ Caspase-3/7 green detection reagent (Invitrogen) was used to for detection after at a concentration of 4 μM according to manufacturer’s protocol.

**NK expansion and labelling**

The purity of the isolated cell suspension was analyzed by flow cytometry using a panel consisting of anti-CD45-APC (Invitrogen, Karlsruhe, Germany), anti-CD56-BV421 (BD Bioscience, San Diego, CA), anti-CD3-BV510 (BD Bioscience), anti-CD16-PE (Biolegend, San Diego, CA) antibodies, 7-AAD (Biolegend) live/dead exclusion and FACS Canto II (BD Bioscience). Expansion was only started with a purity of >90% NK cell enriched fraction on day 0. NK cells were activated and expanded as described previously (28). Briefly, 2x10^6^ cells/ml were cultured in NK MACS Medium (Miltenyi Biotech, Bergisch Gladbach, Germany) supplemented with 5% human plasma (DRK Blutspendedienst) and 1% penicillin/streptavidin. To activate NK cells, IL-15 was added at 10 ng/ml every 3 to 4 days for 15 days before experiments were performed. Staining with CellTrace™ Violet/far red cell proliferation kit (Invitrogen) was performed according to manufacturer’s manual, using a final staining concentration of 3 μM.

**RNA isolation and qRT-PCR**

For transcriptional analysis of 2D versus 3D tumor culture methods, mRNA was isolated according manufacturers protocol using peqGOLD MicroSpin total RNA kit (VWR, Darmstadt, Germany). For normalization between samples 1 μg of mRNA was used for cDNA synthesis, using Revert Aid H Minus first strand cDNA synthesis kit (Thermo Fischer, Waltham, MA, USA). qRT-PCR was performed using SYBR green PCR master mix (Life Technologies) on a 7900HT fast real time PCR system (Applied Biosystems, Waltham, MA, USA) with following oligonucleotides: MCL-1 (fwd: aagccaatgggcaggtct, rev: tgtccagtttccgaagcat), BCL-2 (fwd: AGTACCTGAACCGGCACC, rev: GCCGTACAGTTCCACAAAGG), BCL-xL (fwd: CCCAGGGACAGCATATCA, rev: AGCGGTTGAAGCGTTCCT), BIM (fwd: catcgcggtattcggttc, rev: gctttgccatttggtcttttt), NOXA (fwd: ggagatgcctgggaagaag, rev: cctgagttgagtagcacactcg), BMF (fwd: gagactctctcctggagtcacc, rev: ctggttggaacacatcatcct), PUMA (fwd: GACCTCAACGCACAGTACGA, rev: GAGATTGTACAGGACCCTCCA), BAX (fwd: agcaaactggtgctcaagg, rev: tcttggatccagcccaac) and BAK (fwd: cctgccctctgcttctga, rev: ctgctgatggcggtaaaaa). As internal control three reference genes were used 28S rRNA (fwd: TTGAAAATCCGGGGGAGAG, rev: ACATTGTTCCAACATGCCAG), RPII (fwd: GCACCACGTCCAATGACAT, rev: GTGCGGCTGCTTCCATAA) and G6PD (fwd: ATCGACCACTACCTGGGCAA, rev: TTCTGCATCACGTCCCGGA).
