# Supplementary material for: Pediatric multicellular tumor spheroid models illustrate a therapeutic potential by combining BH3 mimetics with Natural Killer (NK) cell-based immunotherapy
Source: Cell Death Discov. 2022 Jan 10;8:11. doi: 10.1038/s41420-021-00812-6 (PMC8748928; doi:10.1038/s41420-021-00812-6)
Supplement: Supplementary file 4 — Supplementary Figure Legends [file 41420_2021_812_MOESM4_ESM.docx]

**Supplementary Figure Legends**

**Supplementary Figure 1. Immunophenotyping of NK cells**

NK cells were enriched from PBMCs using the Easy Sep Human NK cell enrichment kit and purity was assessed by flow cytometry. Starting PBMCs as well as purified NK cells on day 0 or after an expansion phase of 15 days were stained with the indicated antibodies to determine the containing NK cell fraction. The first gate was set on lymphocytes according to their side- and forward scatter properties, followed by doublet exclusion and thirdly, inclusion of alive 7-AAD- single cells. With the fourth gate only CD45+ cells were gated and further analyzed. NK cells are defined by CD56+ and CD3- expression pattern (upper left quadrant, framed). CD16 was used to further determine the activation status of CD56+ CD3- NK cells.

**Supplementary Figure 2. RD cells form compact, tight spheroids**

RD-GFP cells were cultured in 3D at two different densities (5 000 or 10 000 cells/well). Spheroid formation was monitored over 7 days and demonstrated rapid accumulation of cells within a compact tight spheroid. Imaged with two different objectives are shown (magnification 4x, 10x). Scale bar equals 500 μm.

**Supplementary Figure 3. Spheroid formation is a common feature of pediatric cancer cell lines**. RMS cell lines (T174, TE381.T, RD-GFP, RH30-GFP), osteosarcoma cell lines (U2OS, MG63) or neuroblastoma cell lines (SK-N-AS, UKF-NB3) were cultured as spheroids containing 5 000 cells for 7 days. Spheroid formation was assessed by bright-field microscopy. In addition, spheroids of two different primary patient-derived neuroblastoma tumor samples are shown (neuroblastoma-1: 20 000 cells, neuroblastoma-2: 10 000 cells per spheroid). Scale bar equals 500 μm.

**Supplementary Figure 4. Cell death induced by BH3 mimetics in Kym-1 spheroids.** Kym-1 cells were cultured as 3D spheroids for 3 days before exposure to A1331852 (0.25 μM) or S63845 (0.03 μM) either alone or in combination for 48 hours. Cell death was assessed by PI staining and HOECHST (Hoe) for 30 min before analysis. A) Representative images are shown. B) PI and HOECHST fluorescence was quantified and expressed as the ratio of PI/HOECHST. Data shown are mean + S.D. (n=3), **: p<0.01. Scale bar equals 500 μm.

**Supplementary Figure 5. NK cell migration into RMS spheroids and killing is affected by E:T ratios and spheroid size.** A) Count of CellTrace™ Violet stained NK cells within RH30-GFP spheroids at different E:T ratios. Single cells are determined by size and CellTrace™ Violet positivity. B-C) Illustration of growth inhibiting effect of different E:T ratios of NK cells co-cultured with RMS spheroids as determined by quantification of GFP fluorescence of RH30-GFP (B) or RD-GFP (C) spheroids over a time course of 5 days post NK cell addition (see Figure 6A for experimental setup). Data shown are mean + SEM of 4-6 individual donors (n=4-6), ns: p>0.05, *: p<0.05, **:p<0.01, ***: p<0.001. D-E) Graphs of different sized RD-GFP (D) or RH30-GFP (E) spheroids co-cultured with NK cells at constant E:T ratio. Data shown are mean + SEM (n=3).

**Supplementary Figure 6. BH3 mimetics do not induce cell death in activated NK cells nor alter their cytotoxic and migratory capacity.** Activated NK cells were treated with different concentrations of the BH3 mimetics S63845, A1331852 or ABT199 for 24 hours before analysis of cell death by PI uptake and microscopy. Data shown are mean + S.D. of three individual donors (n=3). B-C) NK cells were pre-treated with S63845 (0.3 μM) or A1331852 (0.3 μM) for 24 hours before co-cultivation with RH30-GFP or RD-GFP spheroids for 5 days. B) The growth inhibiting effect of NK cells was not altered by pre-treatment with BH3 mimetics. C) Migration of CellTrace™ FarRed stained NK cells into RH30-GFP or RD-GFP spheroids within the initial 8 hours of co-culture was not changed by pre-treatment of the NK cells with BH3 mimetics. Data shown are mean + SEM (n=3), **: p<0.01, ***: p<0.001.

**Supplementary Figure 7. ABT-199 does not influence NK cell-mediated killing of RMS spheroids.** Spheroids were generated and treated with BH3 mimetics and activated NK cells as outlined in Figure 6A. Addition of the Bcl-2 inhibitor ABT-199 (3 μM) does not affect NK cell-mediated killing of spheroid as assessed by loss of GFP fluorescence over time. Data shown are mean + S.D. (n=3), ns: p>0.05

**Supplementary Figure 8. S63845 and NK cells reduce growth of neuroblastoma spheroids.** SK-N-AS cells were grown as spheroids and treated with S63845 (3 μM) and NK cells according to the experimental setup displayed in Figure 6A. GFP fluorescence indicative of spheroid size was quantified by microscopy. A) Representative images displaying GFP-labelled spheroids. B) Quantification of GFP fluorescence intensity assessed by microscopy over time. Data shown are mean + S.D. (n=3), ns: p>0.05, **: p<0.01, ***: p<0.001. Scale bar equals 500 μm.

**Supplementary Figure 9. BH3 mimetics do not synergize with NK cells to kill non-malignant HS5stromal spheroids.** A) HS5 spheroids were pre-treated with S63845 (0.3 μM) or A1331852 (0.3 μM) for 4 hours, after which NK cells were added. After 5 days of NK cell co-cultivation spheroids were stained with PI as cell death indicator. PI intensity was quantified, data shown are mean + S.D. (n=3), ns: p>0.05. B) Exemplary images of stained HS5 spheroids on day 5 of NK cell co-cultivation. TL: transmitted light channel. Scale bar equal to 500 μm.

**Supplementary Figure 10. Validation of zVAD.fmk and anti-TRAIL antibody on RMS spheroid killing.** RH30-GFP or RD-GFP spheroids were treated with TRAIL alone (100 ng/ml), in combination with zVAD.fmk (50 μM) or an anti-TRAIL antibody (1 μg/ml) for 24 h. A) Exemplary fluorescence microscopic images of RMS spheroids. B-C) Quantification of GFP and PI fluorescence and calculation of the PI/GFP ratio as cell death indicator. TRAIL-induced killing can be blocked by zVAD.fmk or anti-TRAIL antibody co-treatment in both RMS spheroid models. Data shown are mean + S.D. of technical triplicates of one biological replicate (n=1). Scale bar equals 500 μm.

**Supplementary Video Legends**

**Supplementary Video 1.** The formation of the RD-GFP spheroid, with 10 000 cells per spheroid, was assessed by live cell imaging. For the first 24 h every 10 min an image was taken and stitched as a movie using Fiji.

**Supplementary Video 2.** RH30-GFP spheroids (2 500 cells per spheroid) were generated for 3 days. After generation CellTrace™ Violet stained NK cells were added in an E:T ratio of 5:1. The migration of NK cells into the spheroid was assessed for 12 h, with image acquisition every 20 min.

**Supplementary Video 3.** RD-GFP spheroids (10 000 cells per spheroid) were generated for 4 days. CellTrace™ Violet stained NK cells were added in an E:T ration of 5:1. Migration of NK cells was assessed for 12 h, with image acquisition every 20 min.
