# Supplementary material for: Pediatric multicellular tumor spheroid models illustrate a therapeutic potential by combining BH3 mimetics with Natural Killer (NK) cell-based immunotherapy
Source: Cell Death Discov. 2022 Jan 10;8:11. doi: 10.1038/s41420-021-00812-6 (PMC8748928; doi:10.1038/s41420-021-00812-6)

Supplementary Figure 1

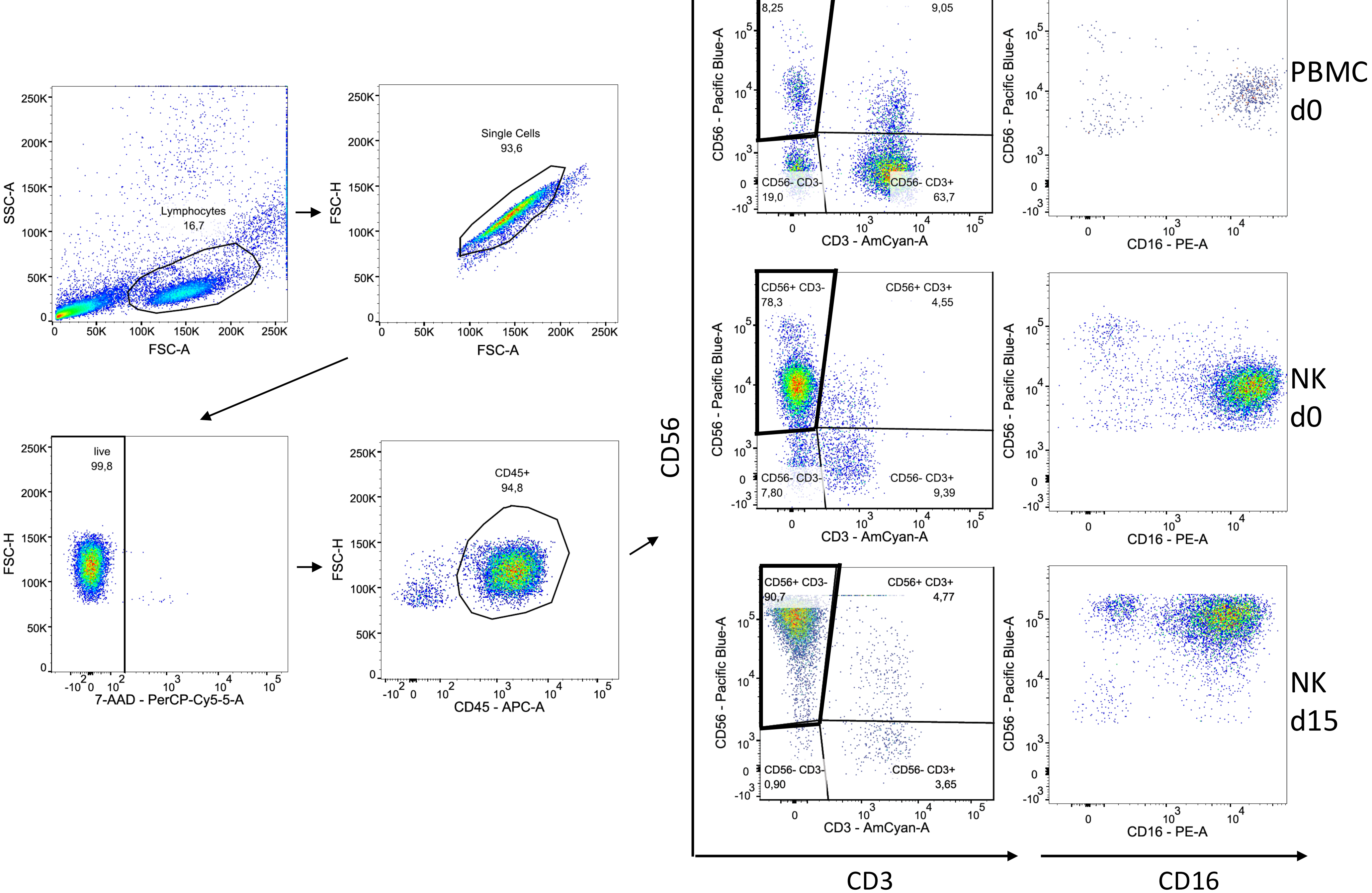

Supplementary Figure 2

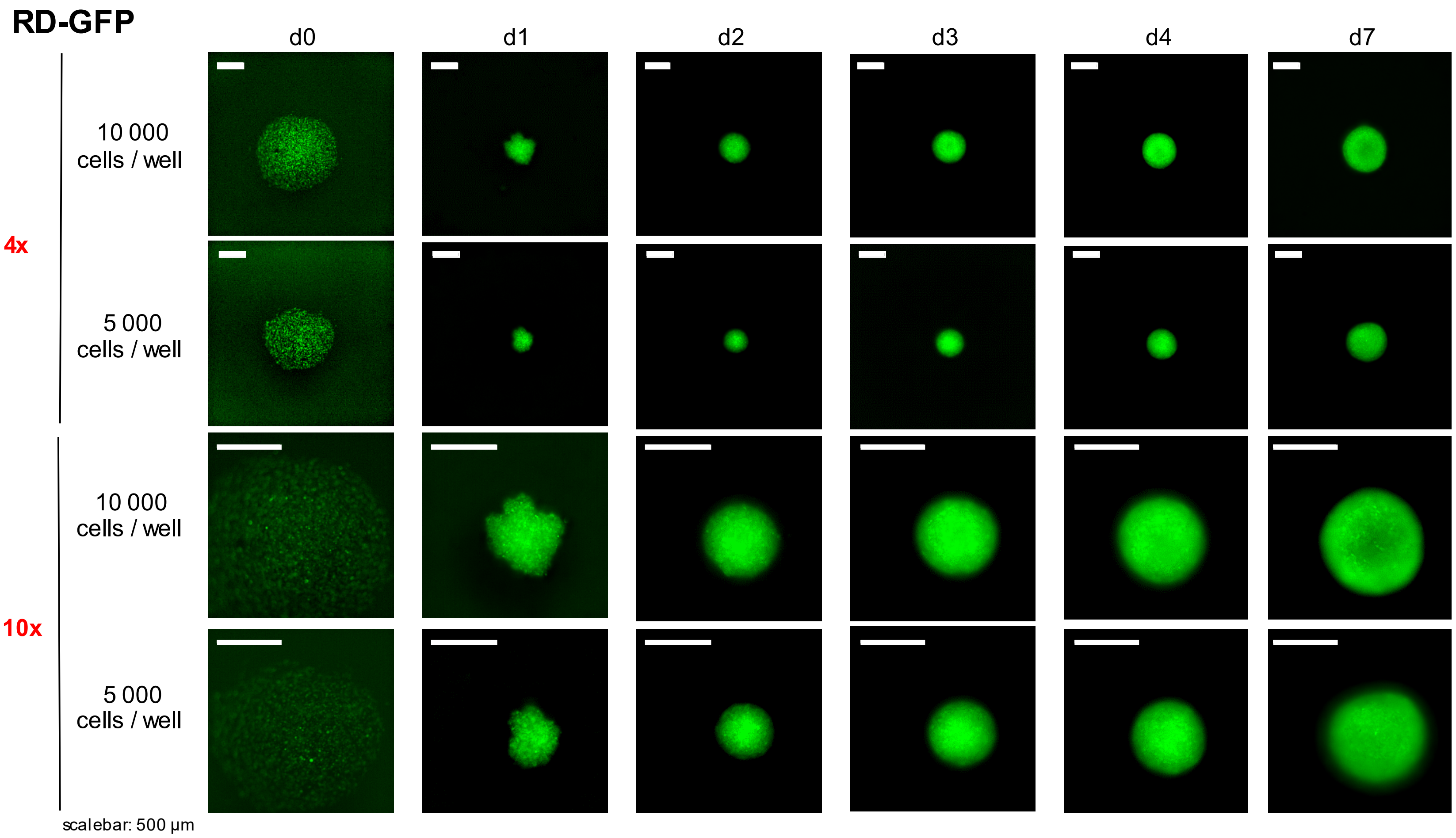

Supplementary Figure 3

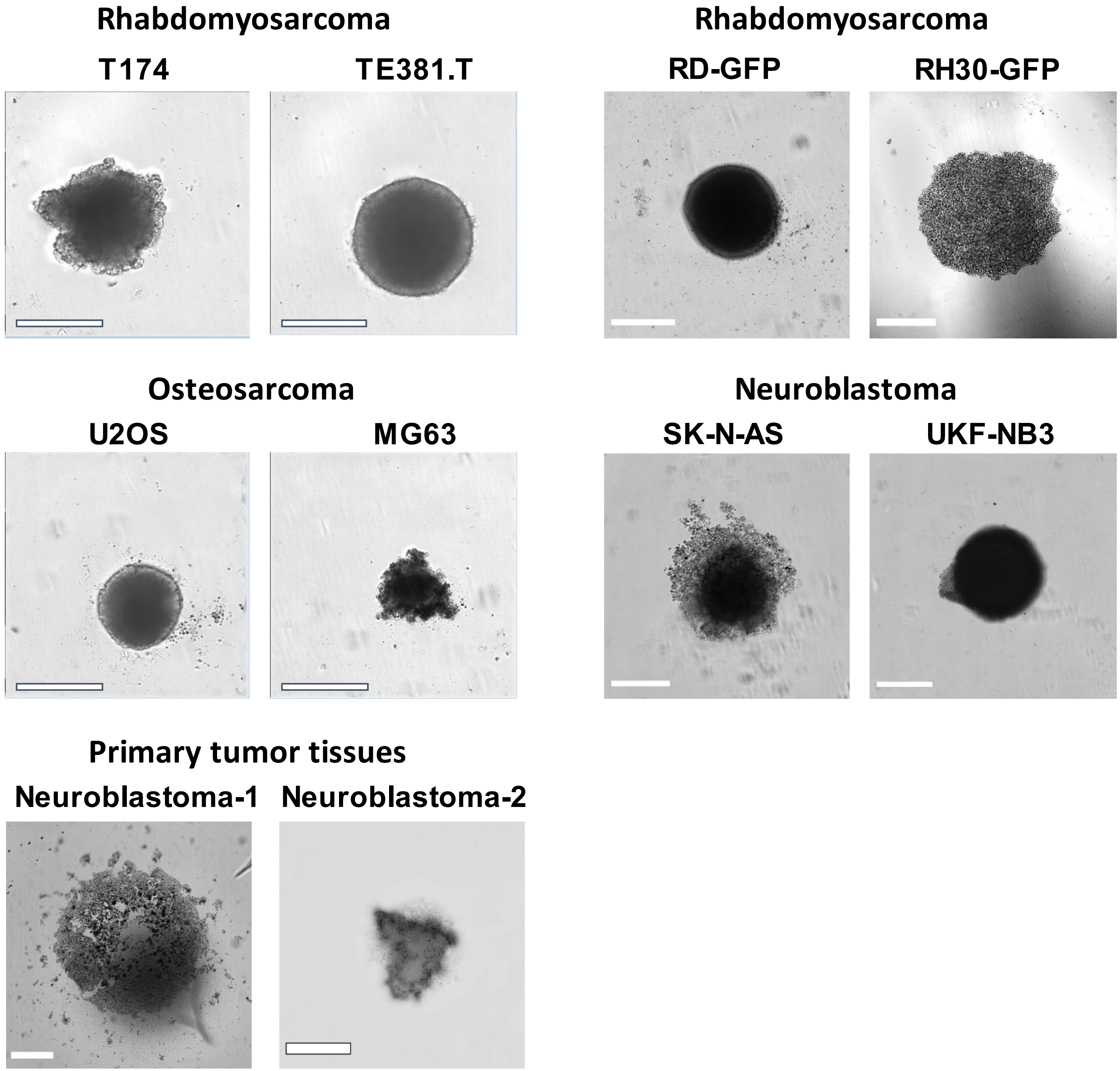

Supplementary Figure 4

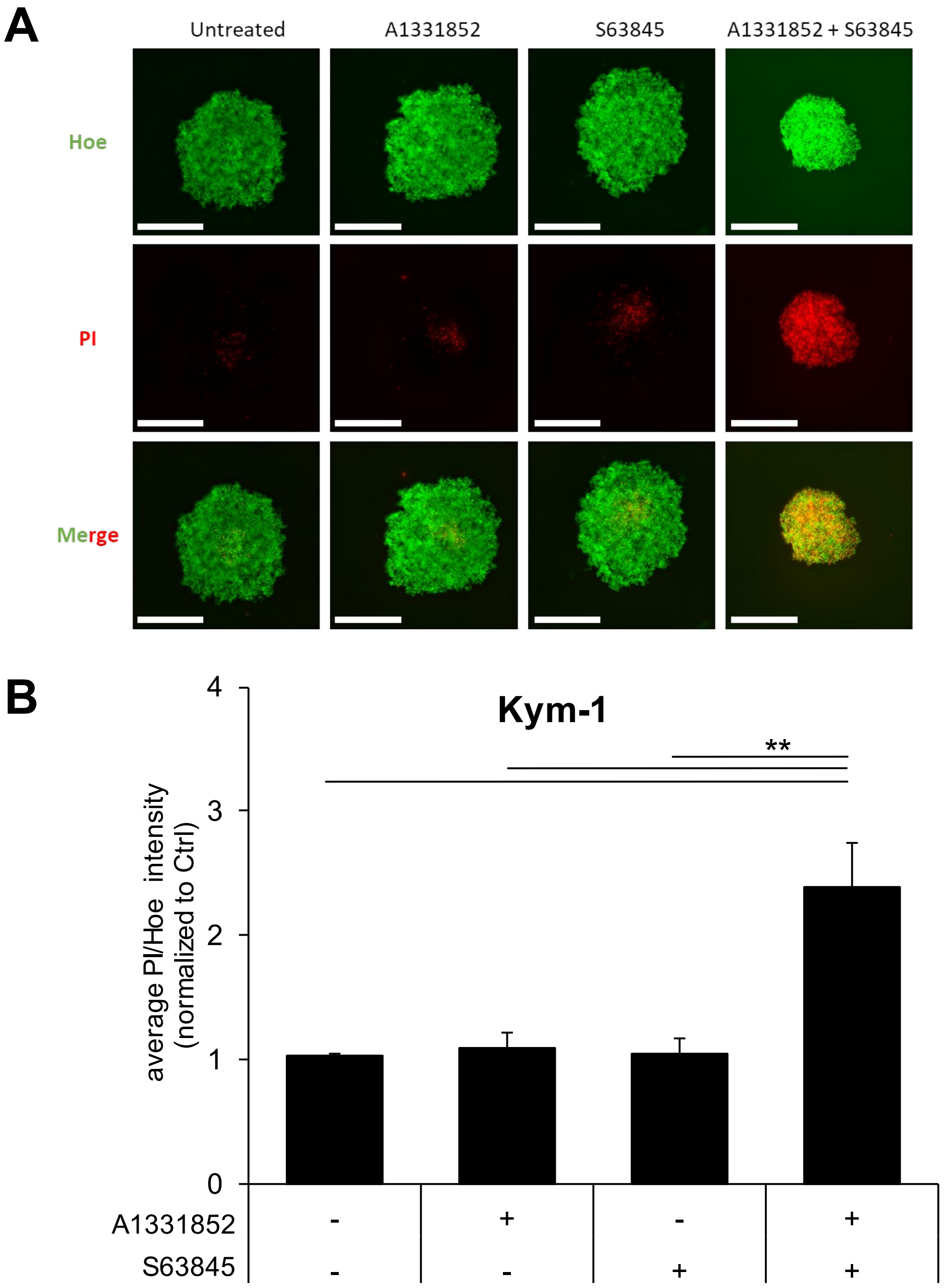

Supplementary Figure 5

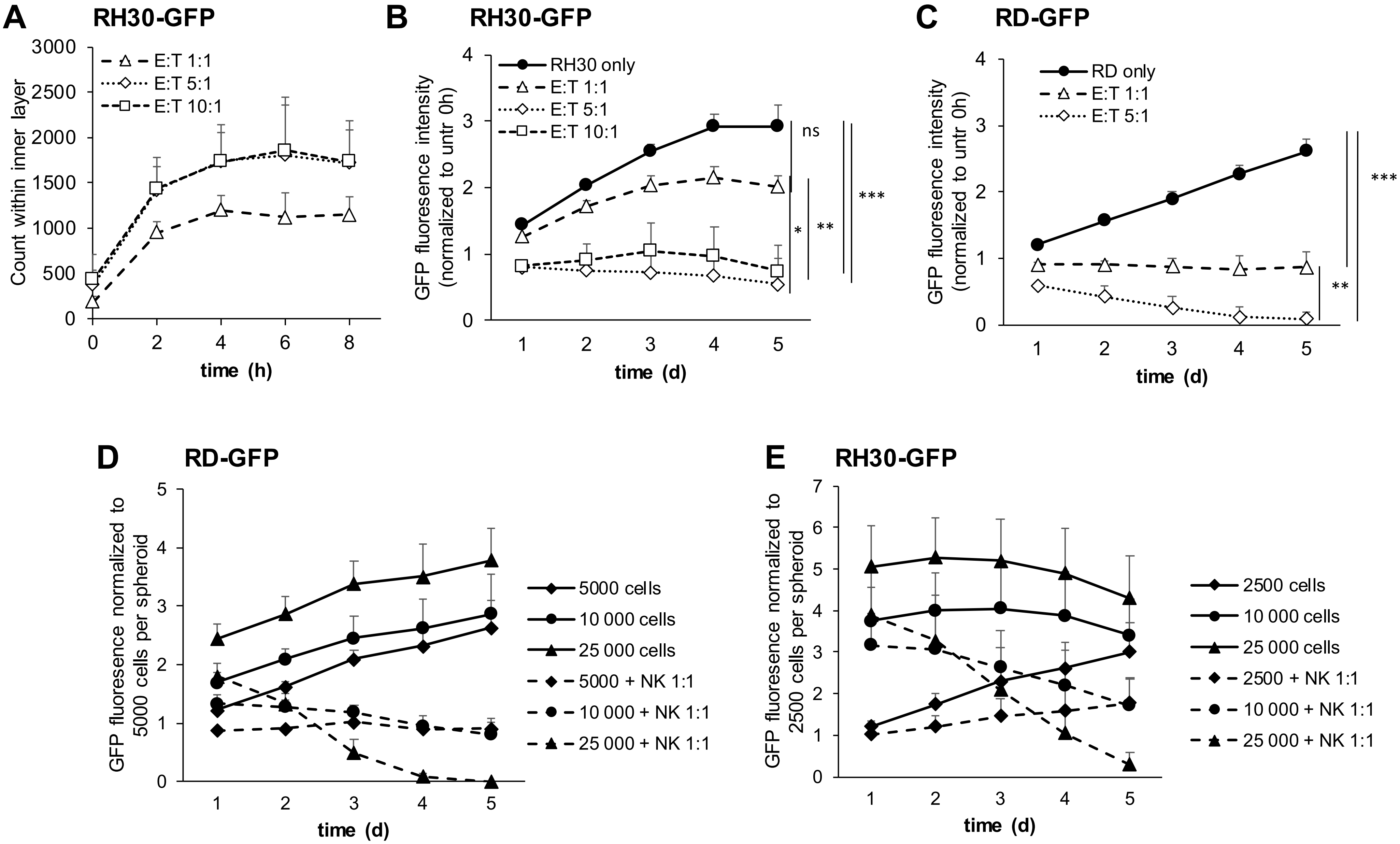

Supplementary Figure 6

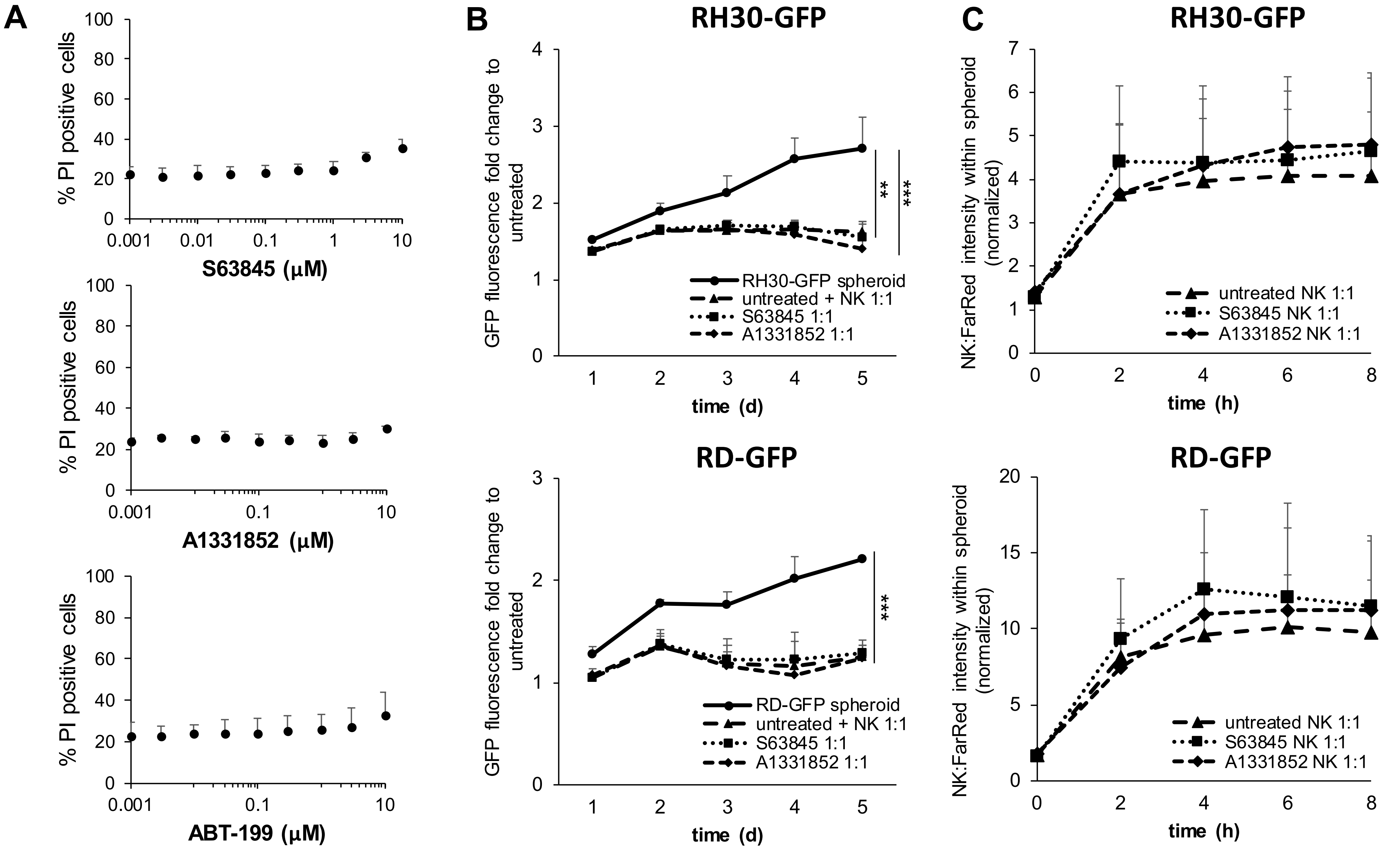

Supplementary Figure 7

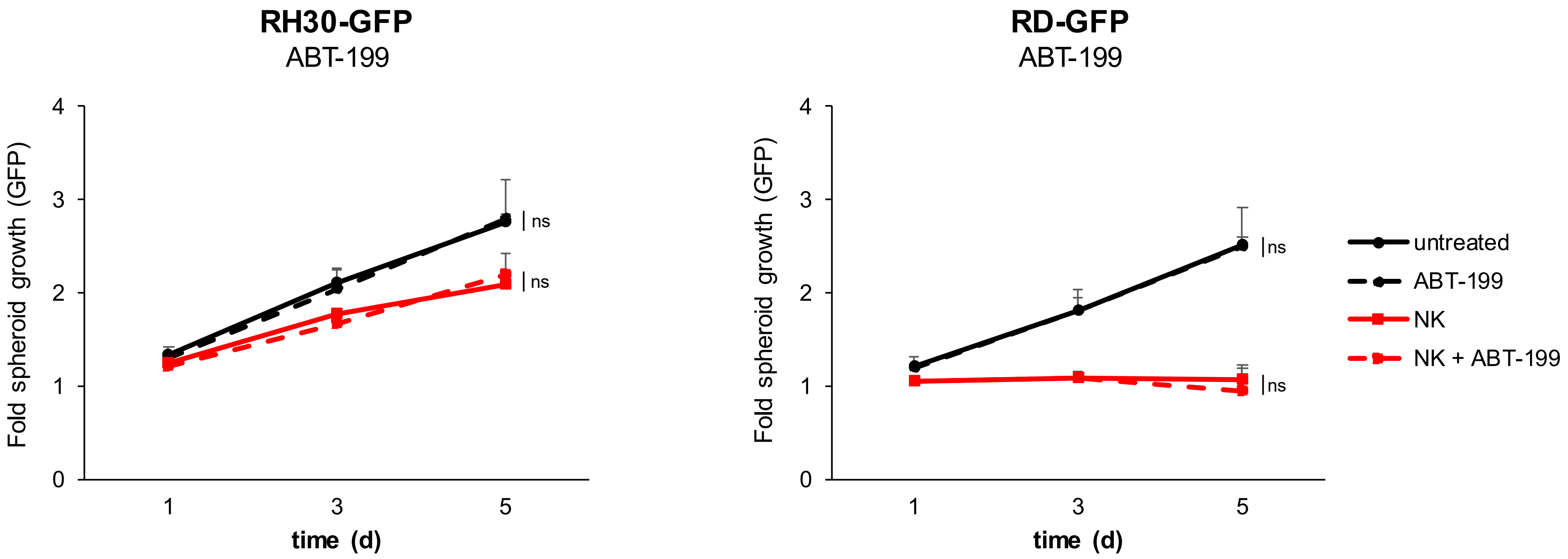

Supplementary Figure 8

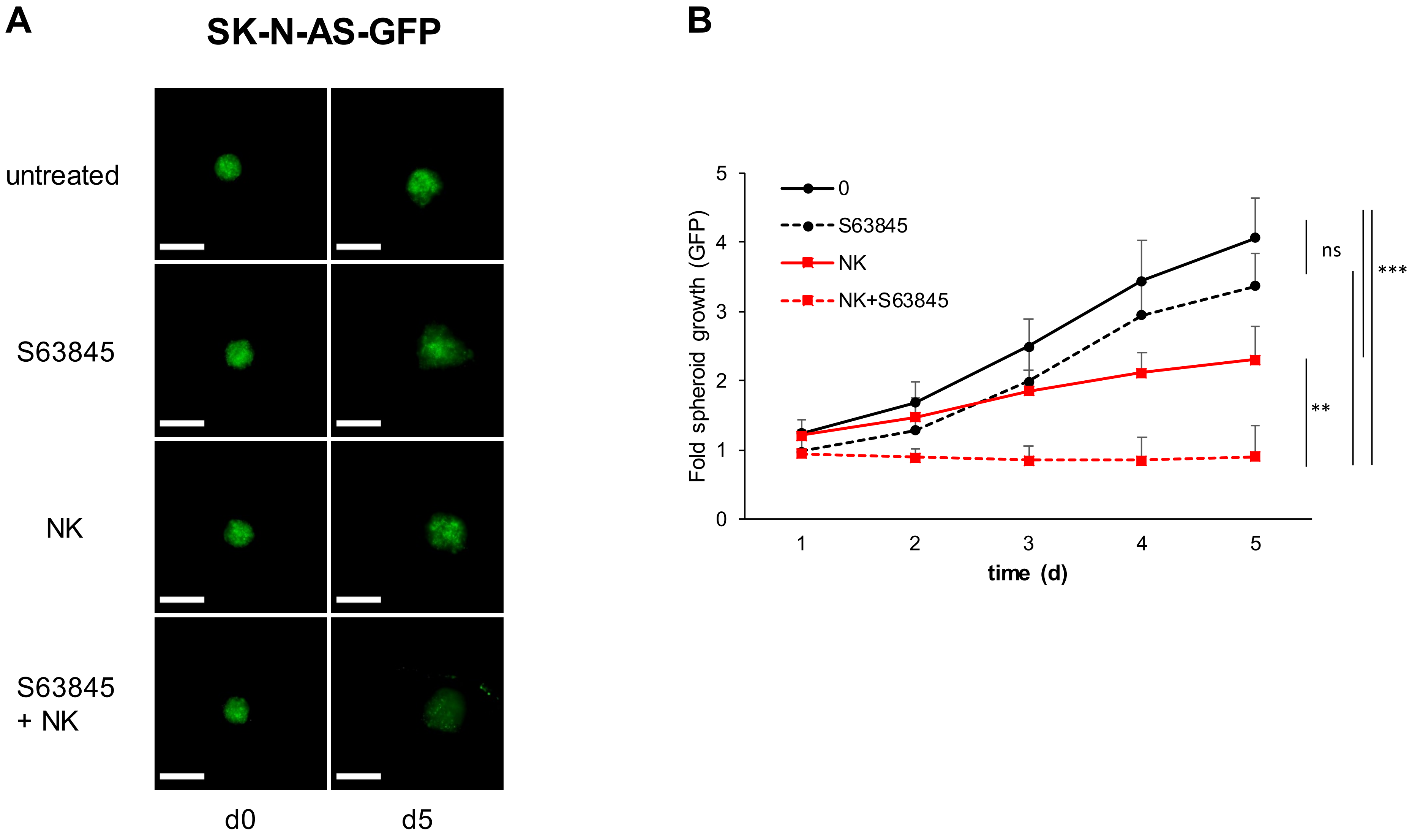

Supplementary Figure 9

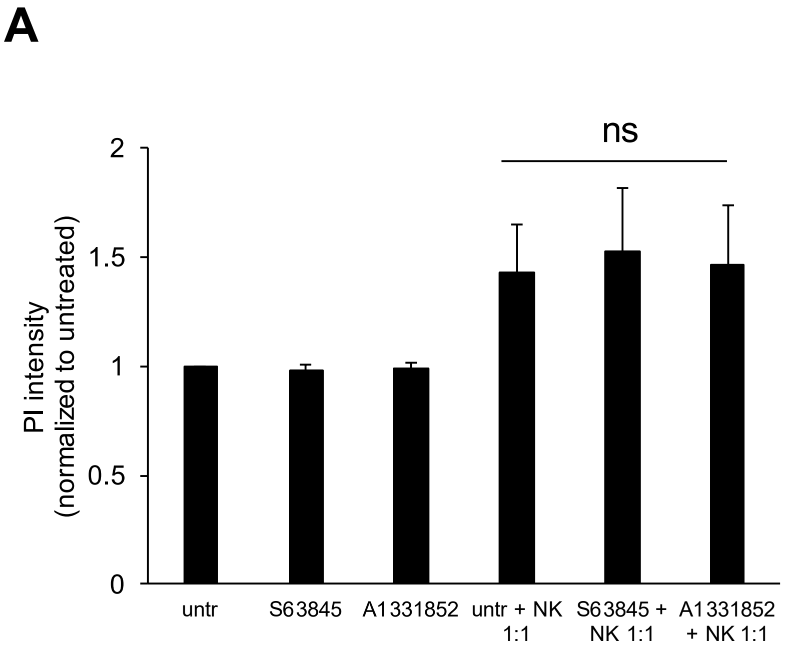

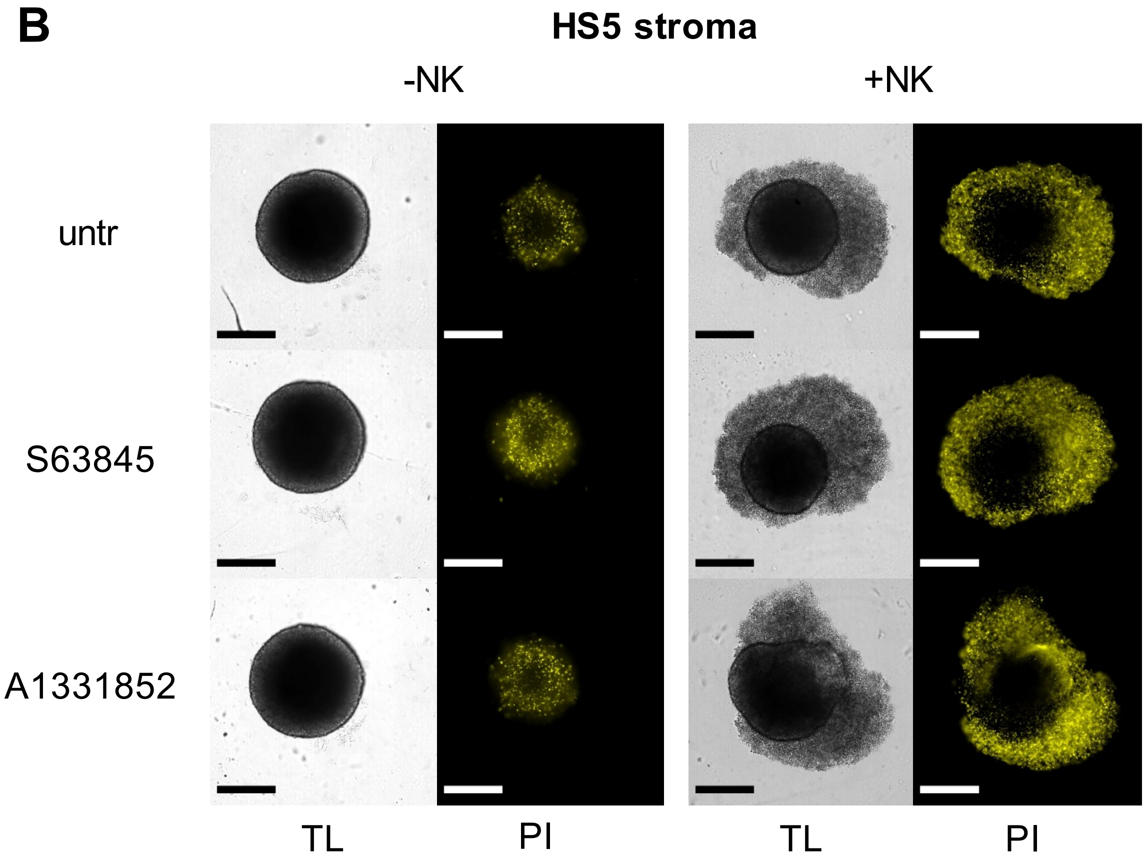

Supplementary Figure 10

A

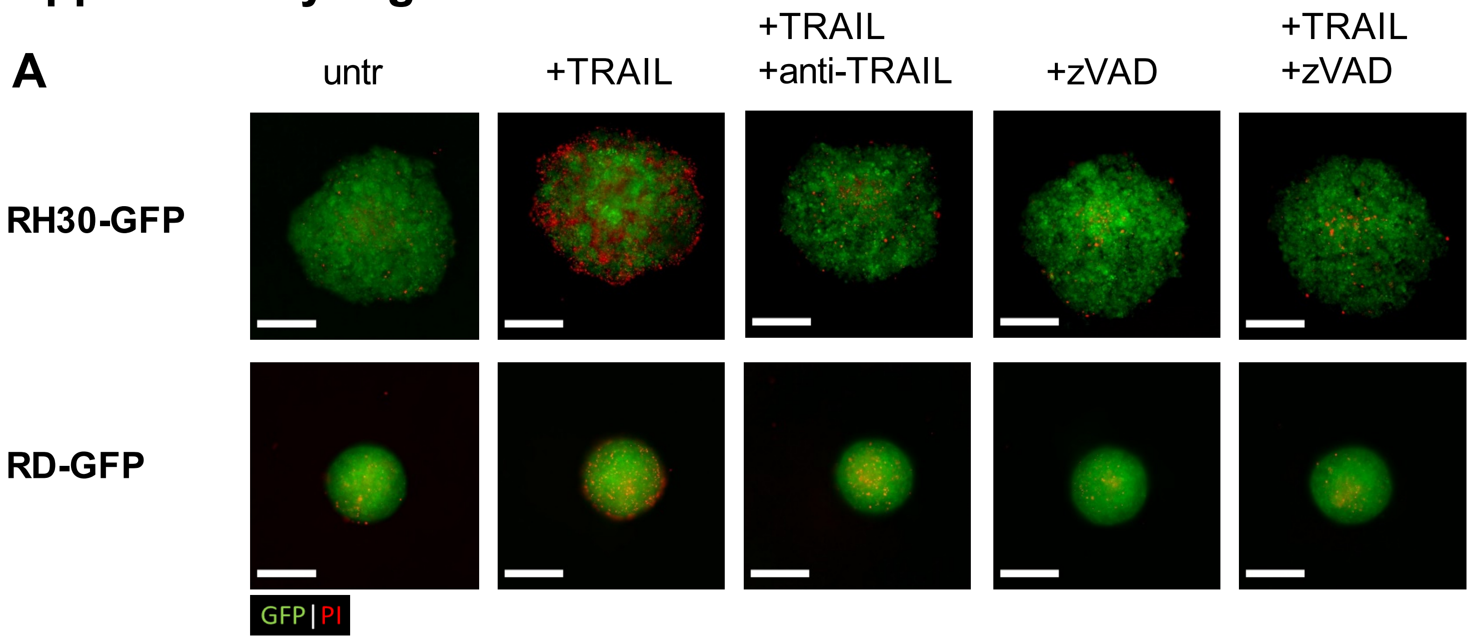

B

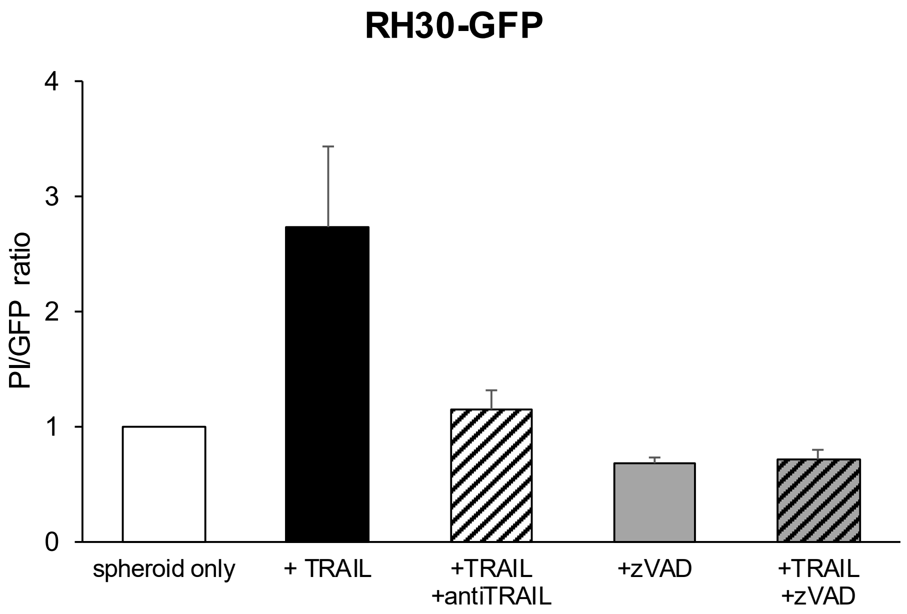

C

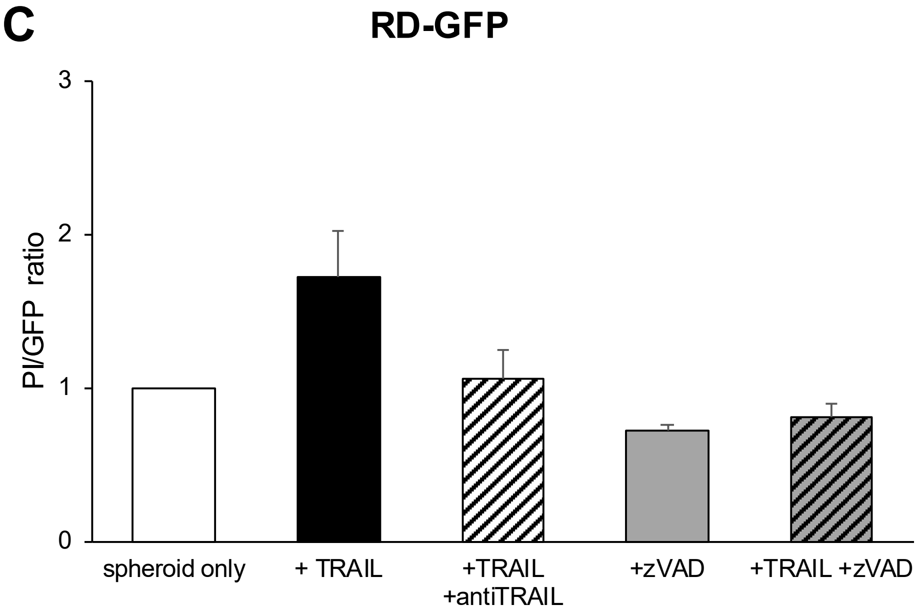

Supplement: Supplementary file 5 — Supplementary Figures [file 41420_2021_812_MOESM5_ESM.pdf]
