# Supplementary figures and images for: Pediatric multicellular tumor spheroid models illustrate a therapeutic potential by combining BH3 mimetics with Natural Killer (NK) cell-based immunotherapy
Source: Cell Death Discov. 2022 Jan 10;8:11. doi: 10.1038/s41420-021-00812-6 (PMC8748928; doi:10.1038/s41420-021-00812-6)

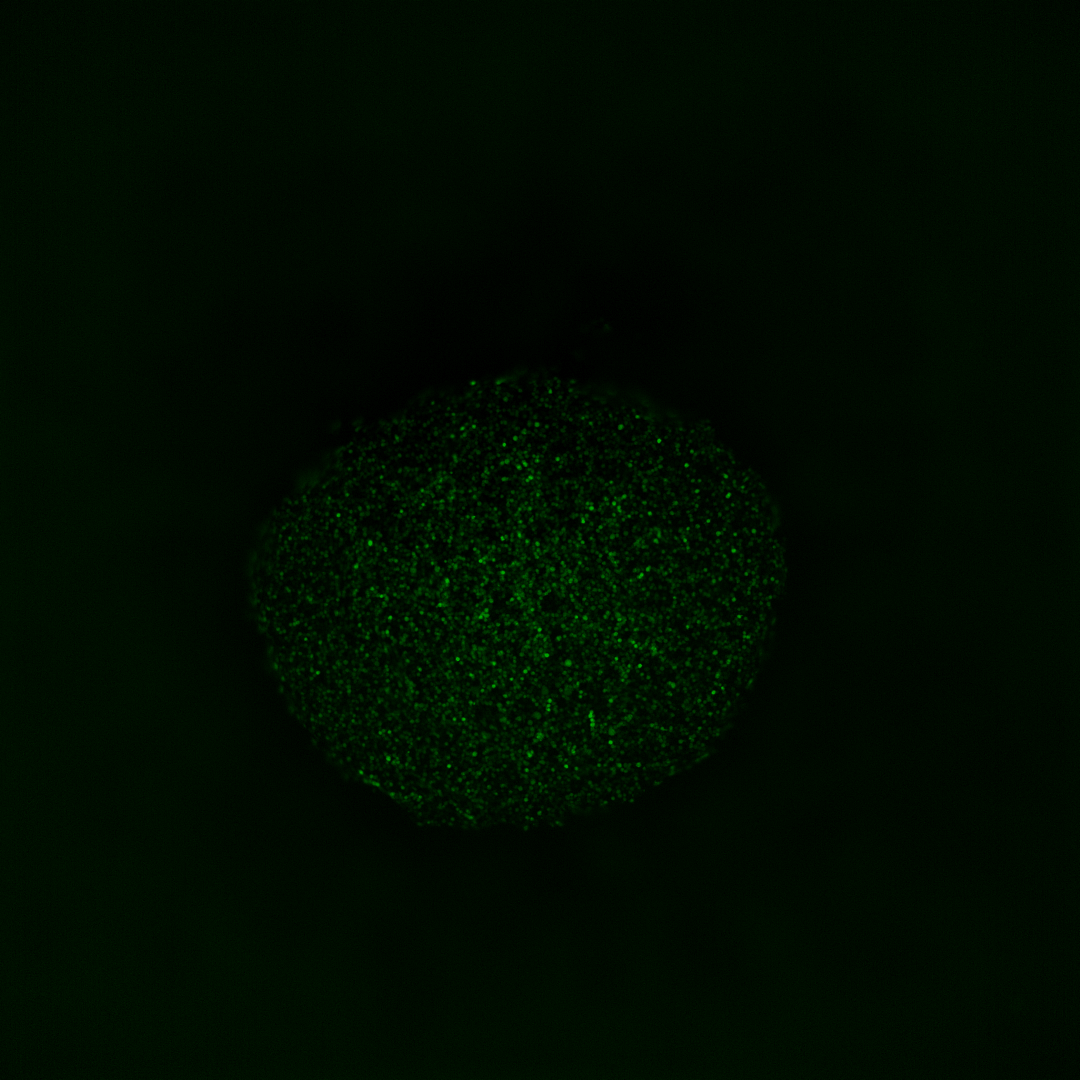

Supplement: Supplementary file 6 — Video 1 [file 41420_2021_812_MOESM6_ESM.gif]

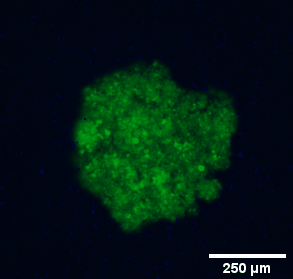

Supplement: Supplementary file 7 — Video 2 [file 41420_2021_812_MOESM7_ESM.gif]

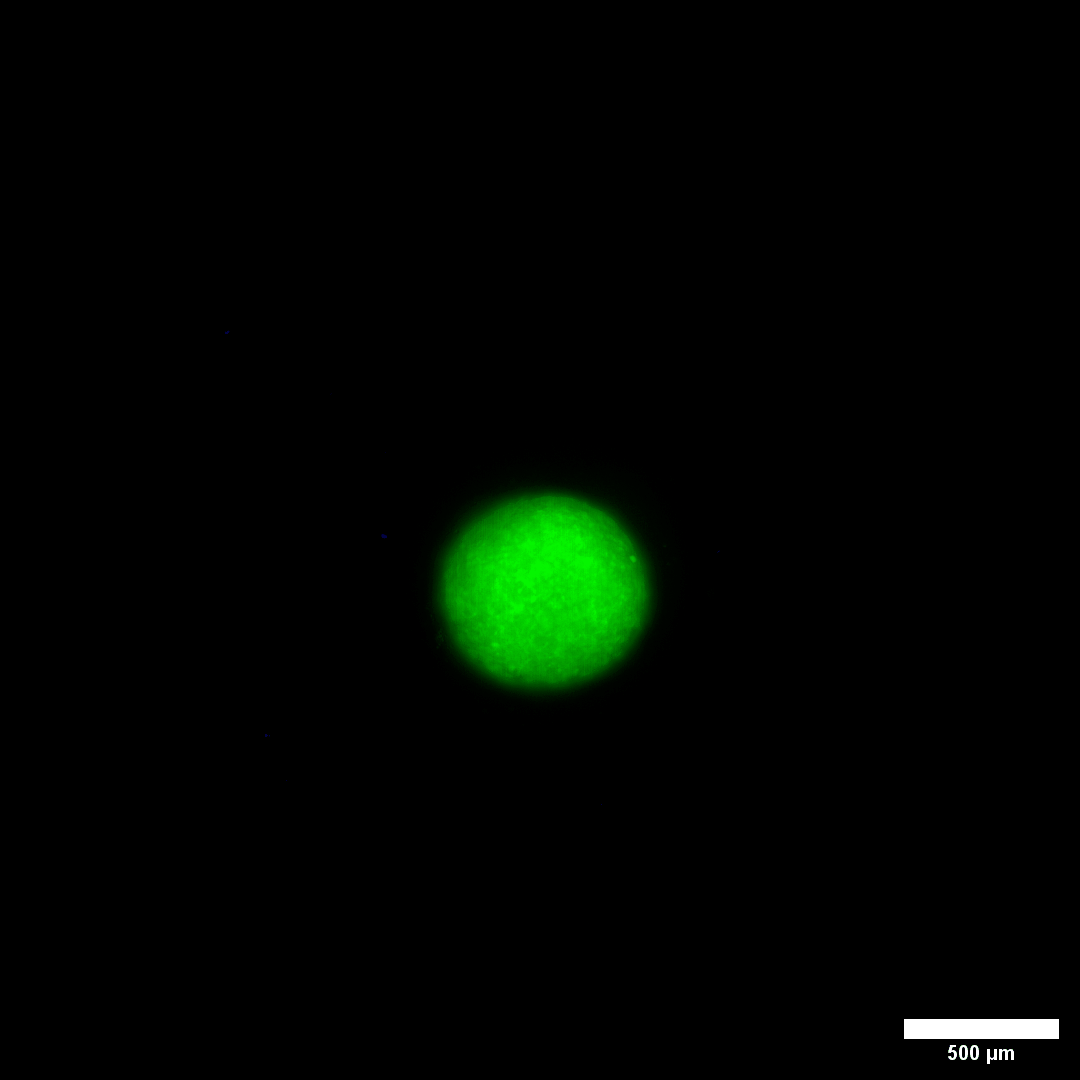

Supplement: Supplementary file 8 — Video 3 [file 41420_2021_812_MOESM8_ESM.gif]
